# Medical Biography

**Published:** 1840-04-01

**Authors:** 


### Medico-Chirurgical Review,

### No. LXIV.

[No. 24 of a Decennial Series.]

JANUARY 1, to APRIL 1, 1840.

### MEDICAL BIOGRAPHY.

I. Memoirs of the Life of Sir Humphry Davy, Bart. LL.D. F.R.S. &c. &c. By his Brother, *John Davy*, M.D. F.R.S. Pp. 475. London, 1839.

II. Biographical Memoirs of the most Celebrated Physicians, Surgeons, &c. &c. By *Thomas Joseph Pettigrew*, F.R.S. &c. &c. Parts 22, 23, 24. Price 3s. each Part.

III. A Memoir of the Life and Character of Philip S. Physick, M.D. By *J. Randolph*, M.D. Philadelphia, 1839.

It is true that we cannot claim Sir Humphry Davy as strictly one of ourselves, or cite his life as the life of a physician. But chemistry is so integral a part of medicine, or, at all events, so linked with it, that its progress touches us deeply, and its professors must as warmly interest us. It is to chemistry, perhaps, that we must mainly look for any material advances in physiology, if not in the practice of physic, and the ties that bind the sciences cannot be drawn too close. These considerations warrant us, we conceive, in turning to the life of Sir Humphry Davy, and in tracing the career of one, who has influenced so materially the fortunes of modern philosophy.

The memoirs before us form the first of four volumes, which, when complete, will contain the collected works of Sir Humphry. The edition promises from the samples before us (we have received the second volume, which consists of Davy's early miscellaneous papers, &c.) to be one of a satisfactory and valuable character; and we have much pleasure in paying, by anticipation, this compliment to the fraternal piety of Dr. Davy, and this act of justice to an editor.

As Dr. Davy observes, the most important part of the history of a man of science is necessarily recorded in his works. But a very interesting part, and a not uninstructive part, is that comprised in his life. It seems a sort of first principle of our nature to be inquisitive about the private history of men, who have made themselves in any way conspicuous. There is a sort of curiosity to learn if those who have soared above common mortality in public life, have shewn themselves different from it in domestic life—have felt its charities, betrayed its weaknesses, and ate, and drunk, and slept, and talked, as ordinary people do. This contrast between the public and the private man is wanted to complete the picture. And it must be owned that it often tends to reconcile the humble with their lot; and to shew that those qualities which conduce, beyond all others, to human happiness, moderation of desires and prudence of conduct, are far from necessary accompaniments of genius. But we turn to Sir Humphry Davy.

He was the eldest son of Robert and Grace Davy. His native place was Penzance, on the shore of the Mount's Bay in Cornwall. He was born on the 17th of December, 1778, at five o'clock in the morning, as is certified in the cover of a large family Bible, in the handwriting of his father. He was christened on the 22nd of January of the following year, and was nursed by his mother. He was a healthy, strong, and active child, and in every respect forward. We are not told of any lambent flame playing round the infant's cradle, but the baby philosopher talked early, walked early, had a wonderful memory, and, like Pope, he—

"Lisped in numbers, for the numbers came."

How much, not of what we *know*, but of what we feel, and do, may be traced to school and school-boy days. A "public-school-man" may generally be distinguished from a "private-school-man" or a "private-tutor-man," by his confident manner and bolder bearing. How many a gallant spirit has been cowed by the pedagogue's petty tyranny—how many a noble mind has been cramped by the cold formality of the—

"Dull dry lesson daily got by rote."

It has been said that the character of a nation depends upon its mothers. It is only an extension of the same idea to assert that a nation's character depends upon its school-masters. For the school-master carries on the mother's work, and, surely, at a still more momentous epoch of our history. What bigotry, what false respect for forms, what hatred of innovation, what prejudice for obsolete ideas, may not we owe to our school-masters. Those who are to educate the nation, the nation should first take care to educate.

The first school that Master Humphry was sent to, was that of a Mr. Bushell, at which reading and writing only were taught. This master, then an old man, remarking the rapid progress of his young pupil, in a very disinterested manner, recommended the removing him (he was then six years old) to the grammar-school. This school was kept by the Rev. Mr. Coryton, a man of irregular habits, and ill-fitted for the office of teaching youth, and as deficient in good method as in sound scholarship. He was generally careless, indiscriminating, and indulgent in regard to the manner in which the boys performed their exercises; but occasionally severe, acting the tyrant, and punishing heavily slight offences. Pulling the boys' ears was practised by him in the most capricious manner, and Davy was too frequently a sufferer from this infliction. It is recorded of him that, on one occasion, he appeared before Mr. Coryton with a large plaster on each ear, and that, when asked by his master what was the matter with his ears, he replied, with a grave face, that he had "put the plasters on to prevent a mortification." Yet, in after-life, he was inclined to believe that Mr. Coryton had done him no harm. In a letter to his mother, written in 1802, he makes some inquiries respecting his brother, then at school at Helston, and asks—"Does John like Latin and his school, now the novelty of the first impression is passed away? I recollect I was rejoiced when I first went to Truro school, but I was much more rejoiced when I left it for ever. Learning naturally is a true pleasure: how unfortunate then it is that in most schools it is made a pain? Yet Dr. Cardew comparatively was a most excellent master. I wish John may have as good a one. After all, the way in which we are taught Latin and Greek does not much influence the important structure of our minds. I consider it fortunate that I was left much to myself when a child, and put upon no particular plan of study, and that I enjoyed much idleness at Mr. Coryton's school. I perhaps owe to these circumstances the little talents that I have, and their peculiar application. What I am I have made myself; I say this without vanity, and in pure simplicity of heart!" And we dare say that Davy was right. His biographer communicates some interesting particulars of his boyish habits.

"During the early part of his life, to which he thus refers the formation of the bias of his character, he was more distinguished out of school and by his comrades than for any great advance in learning. Within school the stimulus was wanting to exertion. He appears to have taken the lead in his class, and to have been satisfied; or rather, as may be inferred from what has been mentioned, disgusted, with the uninviting form in which classical knowledge was offered to him, and the repulsive circumstances accompanying it. From his facility in composing Latin and English verse, his assistance was often requested, even by boys much older than himself, in these exercises; and in writing valentines and love-letters he shone so pre-eminently, and gave his aid so willingly, that he is said to have been generally resorted to on all emergencies of boyish loves. Another cause of popularity amongst his comrades was his power of diverting by telling them stories; and so attractive were the stories, commonly of wonder and terror, which he related, that they were in the habit, in an evening, of collecting at a particular place to wait for him, as under the balcony of the Star Inn, which afforded shelter, and where, if there happened to be a cart, he would get into it, and hold forth to his young audience. His stories, greatly embellished by his invention, were collected partly from books, especially the 'Arabian Nights,' of which he was ever very fond, and partly from old people, with whom he was a great favourite, particularly from his grandmother Davy;—a woman of a fervid and poetical mind, of a retentive memory, and who had at command a rich store of traditions and marvels. His other boyish tastes and pursuits, like the preceding, followed him into manhood. Fishing was very early a favourite amusement of his; indeed, his taste for it appears to have been almost instinctive. When a child he used, with a crooked pin tied to a stick by a bit of thread, to go through the movements of the angler, and fish in the gutter of the street in which he lived. The earliest indication that I am aware of, which he showed of his fondness for experimenting, for which he was afterwards so distinguished, was in making fire-works. My eldest sister very well remembers, that she was his assistant in this undertaking, and that their workshop was an unfurnished room, in which, in bad weather, the Rev. Dr. Tonkin (the elder brother of Mr. John Tonkin, his early benefactor), then advanced in age, and a valetudinarian, took exercise on his chamber-horse, a large arm-chair attached to spring boards, which boards served for a table for compounding the ingredients of the squibs and crackers." 6.

In the lad with "crooked pin and thread" might be seen by those who could read the hieroglyphic page of human character, the enthusiastic author of a future Salmonia, and the squibs and crackers were the earliest experiments of the neophyte discoverer of potassium. Amidst all this fun, with St. Michael's Mount and the deep blue sea before him, the boy who seemed so thoughtless was cherishing that ardent love of Nature which appears to be a very part of genius; at least of that genius which yearns after philosophy. He roamed amidst the romantic scenery around him, carrying with him his fishing-rod and gun, and interrogating Nature in this sportsman fashion. He was a gardener too, and collected and painted birds and fishes, many, no doubt, of his own killing. To those about him Humphry was no prodigy. Many such another boy has lived, and died, and done nothing for the world, and the world nothing for him. The genius was there, as it was in Davy—for that genius to bear fruit, circumstances must shine upon it.

Davy went to Dr. Cardew's school when fourteen years old, on the 15th of January, 1793. He left it in December of the same year, and, at the age of fifteen, his school education was finished, his self-education about to begin. And it began with a time of trial. Like one of his own birds, he hovered for awhile round the tree of science before he settled on it. He returned to Penzance, rode and fished, and strolled even more than before, and bade fair to be a denizen in the castle of indolence. But his father's health declined, and in December 1794, he died. This must have brought serious thoughts to the son's mind, thoughts, perhaps of time misspent, of an uncertain future. He chose a profession (our own), and on the 10th of February 1795 (the date of his indenture), he was apprenticed to Mr. Bingham Borlase, a man of talent, then practising as a surgeon and apothecary in Penzance, who afterwards received a diploma, and was distinguished as a physician.

And now the energy which sport had formed, or rather which had broken out in sport, was applied in earnest. His note-books were commenced, and the earliest of them, bearing the date of this year, is, in every respect curious. It is a small quarto, with parchment covers: on one of the covers, on the outside, is the figure of an ancient lyre, drawn with his pen, and, on the other, an olive wreath encircling a lamp; as if in anticipation of his great discovery of confining flame in the safety-lamp. At the commencement of it is the following plan of study.

1. Theology.

   Or Religion, } - { taught by Nature.

   Ethics, or moral virtues } - { by Revelation.

2. Geography.

3. My Profession.

   1. Botany.

   2. Pharmacy.

   3. Nosology.

   4. Anatomy.

   5. Surgery.

   6. Chemistry.

4. Logic.

5. Language.

   1. English.

   2. French.

   3. Latin.

   4. Greek.

   5. Italian.

   6. Spanish.

   7. Hebrew.

6. Physics.

   1. The doctrines and properties of natural bodies.

   2. Of the operations of nature.

   3. Of the doctrines of fluids.

   4. Of the properties of organised matter.

   5. Of the organisation of matter.

   6. Simple Astronomy.

7. Mechanics.

8. Rhetoric and Oratory.

9. History and Chronology.

10. Mathematics.

This is pretty comprehensive for an apothecary's apprentice at the Land's End. But that apprentice was a bit of a visionary, and dreamt, we dare to say, some such dreams as bewildered the "last days of a philosopher."

"To give some distinct idea of the bent of his studies at this time, I shall notice briefly the principal topics which appear in this MS. volume. It opens with 'Hints towards the Investigation of Truth in Religious and Political Opinions, composed as they occurred, to be placed in a more regular manner hereafter.' His first essay is 'On the Immortality and Immateriality of the Soul;' the second bears the title of 'Body, organised Matter;' and his third is 'On Governments.' Then there follows :—'On the credulity of Mortals;' next, 'An Essay to prove that the Thinking Powers depend on the Organisation of the Body;' next, 'A Defence of Materialism;' next, 'An Essay on the ultimate End of Being;' next 'On Happiness;' then 'On Moral Obligation.' These topics occupy rather more than one half of the book; the other part, which appears to have been written after, commences at the opposite end, inverted. The subjects treated of occur in the following order :—'Theology;' 'The Christian Religion not repugnant to true Philosophy;' 'An Essay on the Influence of Climate on national Manners and Happiness;' 'On Friendship, an Essay;' and besides these, which are the principal contents of the book, there are some verses, and the beginning of a romance, called '*An Idyl,*' in prose, in the form of dialogue; the characters, 'Trevelis, a warrior, and friend of Prince Arthur, and Morrobin, a Druid; the scene, a 'cliff at the Land's End, in Cornwall; and the time, night.'" 14.

If young Humphry could have written any thing good on any one of all those subjects, he need never have discovered the safety-lamp to give him fame. And if he could have solved but one of his problems, he would have had all the immortality the *world* can offer.

At this time, he was a sceptic in religion, and a sophist in knowledge. He put creeds into syllogisms, decided the question of the immortality of the soul by a formula, and dogmatised in sciences of which he could scarcely know the first principles. With the lapse of years came a change of sentiments, and the man who began as a matter of fact materialist, ended, as all who have read the "Consolations" know, as a Spiritualist of the most sublime description. As a matter of curiosity we subjoin his early argument for, his later one against, materialism. The greater force of truth will be evident on the nobler side.

Let us listen to the bold materialist, at the age of sixteen years and a half.

"If we trace the progress of the thinking powers from their original source, we shall find that they owe their being to perception. A child, when it first comes into the world, is without ideas, and consequently, he does not think. All the actions he performs arise from instinct. When hunger calls him he satisfies his cravings with the milk of his mother; nor does he at all differ from the most stupid animal, only in being more helpless. He possesses but a small degree of perception; his attention is awakened with difficulty; the memory is weak and faint; and the ideas, without being often repeated, are not retained. As the child advances in years, the nerves become firmer and the brain stronger; perception is quicker, and the memory is more tenacious and retentive. Judgment, the result of perception and memory, is displayed : by degrees reason as slowly advances; and, lastly, disposition, the boundary of human intelligence, appears. Gradual is the progress of mind from sense to science. When the mental faculties have reached their highest perfection in manhood, they gradually decline; and nought is left of all the wreck of human knowledge but pure sensation, a principle gradually decaying with the falling frame. From hence there follows a self-evident corollary, that the thinking powers are not always the same: whatsoever is not always the same is naturally changeable, is mortal and material. Besides, we have traced the power of thinking from 0 to 0, increasing with the corporeal powers, and decreasing and ending with them." 16.

Nineteen years and a half afterwards, he wonders at his own presumption and in the same note-book, he combats materialism thus:—

"1. The power of thinking does not naturally belong to matter.

2. Motion, if ever so artfully distributed, it is plain, can produce nothing but motion.

3. Matter acts only in proportion as it moves; thinking is acting without motion; *ergo*, that which thinks is not matter.

4. The universality of the hypothesis.

5. Internal consciousness of the existence of a monadic indivisible soul." 17.

After quoting some further heads of religious arguments or essays, his biographer observes:—It is interesting to compare these his early inquiries on the subject of religion with those he engaged in at a later period, as expressed in his "Salmonia," and "Consolations in Travel." We may trace in the former the germs of many of the latter; and, indeed, the resemblance is often so marked, that the trains of thought have very much the character of recollections: with this marked difference, however, that in youth he considered reason as all-sufficient, whilst in later life he mistrusted it, as inadequate, and built his faith on internal or instinctive feeling, rather than on any process of ratiocination.

And thus, we believe, it will ever be. The forms of reasoning satisfy the mind in youth, because they seem adapted to the discovery of truth, and capable of eliciting it. But frequent disappointment damps our confidence, our syllogisms often fail and not uncommonly deceive, despondency or scepticism takes the place of confidence in cold temperaments, whilst faith and a trust in innate feelings possess enthusiastic ones.

Young Humphry was a poet. What ardent mind has not loved poesy, has not attempted it? His biographer introduces a rather lengthy piece, which bears for its title;—The Sons of Genius. We discern in it that "longing after immortality," which the boy-philosopher felt, almost before he knew it. We shall quote some of the stanzas partly as samples of his poetic powers, partly as evidence of the state and the progress of his character.

"While superstition rules the vulgar soul,
Forbids the energies of man to rise,
Raised far above her low, her mean control,
Aspiring genius seeks her native skies.

She loves the silent, solitary hours;
She loves the stillness of the starry night,
When o'er the bright'ning view Selene pours
The soft effulgence of her pensive light.

'Tis then, disturb'd not by the glare of day,
To mild tranquillity alone resign'd,
Reason extends her animating sway
O'er the calm empire of the peaceful mind.

Before her lucid, all-enlightening ray,
  The pallid spectres of the night retire;
She drives the gloomy terrors far away,
  And fills the bosom with celestial fire.

Inspired by her, the sons of genius rise
  Above all earthly thoughts, all vulgar care;
Wealth, power, and grandeur, they alike despise,—
  Enraptured by the good, the great, the fair.

A thousand varying joys to them belong,—
  The charms of nature and her changeful scenes:
Theirs is the music of the vernal song,
  And theirs the colours of the vernal plains.

Yet not alone delight the soft and fair,
  Alike the grander scenes of nature move;
Yet not alone her beauties claim their care,
  The great, sublime, and terrible they love.

The sons of nature,—they alike delight
  In the rough precipice's broken steep;
In the bleak terrors of the stormy night;
  And in the thunders of the threatening deep.

When the red lightnings through the ether fly,
  And the white foaming billows lash the shores;
When to the rattling thunders of the sky
  The angry demon of the waters roars;

And when untouch'd by Nature's living fires,
  No native rapture fills the drowsy soul;
Then former ages, with their tuneful lyres,
  Can bid the fury of the passions fall.

By the blue taper's melancholy light,
  Whilst all around the midnight torrents pour,
And awful glooms beset the face of night,
  They wear the silent, solitary hour.

Ah! then how sweet to pass the night away
  In silent converse with the Grecian page,
Whilst Homer tunes his ever-living lay,
  Or reason listens to the Athenian sage.

Like the tumultuous billows of the sea
  Succeed the generations of mankind;
Some in oblivious silence pass away,
  And leave no vestige of their lives behind.

Others, like those proud waves which beat the shore,
  A loud and momentary murmur raise;
But soon their transient glories are no more,
  No future ages echo with their praise.

Like yon proud rock, amidst the sea of time,
Superior, scorning all the billow's rage,
The living sons of genius stand sublime,
The immortal children of another age.

For those exist whose pure ethereal minds,
Imbibing portions of celestial day,
Scorn all terrestrial cares, all mean designs,
As bright-eyed eagles scorn the lunar ray.

Theirs is the glory of a lasting name,
The meed of genius, and her living fire;
Theirs is the laurel of eternal fame,
And theirs the sweetness of the muse's lyre."

Here are certainly the aspirations after philosophy and fame, which, in coming years, were to make both his own.

In 1796, he entered on the study of mathematics. One book is almost entirely confined to this subject; and in it, his progress may be traced through the following branches, which he enumerates under the head of "Mathematical Rudiments;" viz., "Fractions, vulgar and decimal; Extraction of Roots; Algebra (as far as quadratic equations), Euclid's Elements of Geometry; Trigonometry; Logarithms; Lines and Tangents; Tables; Application of Algebra to Geometry," &c. He entered upon Fractions, February 8th, 1796; and he appears to have finished the elementary course which he assigned himself in little more than twelve months, for the last date is January 2, 1797, when he was commencing the eleventh book of Euclid, and he had gone through most of the other branches.

In this study, his biographer informs us, he was very systematic; the propositions are all entered very neatly, and the demonstrations given; the diagrams are invariably done with a pen, without the aid of mathematical instruments, not even of a common compass and ruler, except in one or two instances. This circumstance of itself would show that he engaged in these studies without a master, which was the fact, and perfectly voluntarily on his part, from the conviction of their usefulness preliminary to the study of physical and chemical science.

Davy was very fond of metaphysics, and appears to have been acquainted with the writings of Locke, Hartley, Bishop Berkeley, Hume, Helvetius, Condorcet, Reid and his followers, as well as, in some measure, with the doctrines of Kant and the Transcendentalists. It is rather amusing to read the titles of Davy's papers, of the works that he began—we need not add, that he never finished. Had he only completed one of them, and he seems to have commenced some scores, he would have done the work of a lifetime. At Penzance, for instance, we find him composing "Observations relating to Existence," a pretty comprehensive theme.

If, to parody the well-known epigram, his metaphysics were physic, his physic was very metaphysical. His biographer admits that the professional studies of which he left any memoranda were chiefly theoretical, relating to physiology, in which he pursued a path similar to that he first followed in metaphysics and religion,—being a process of abstract reasoning founded on a few principles, or abstract terms, by which, like Hartley and Brown, he attempted to explain all the phenomena of life. How lucky that he found all this to be fallacious.

In 1797, he seems to have gone in earnest to the study of natural philosophy. The billiard-table had previously given him some ideas, and furnished some not uninteresting experiments on the laws of the collision of bodies. But billiard-balls and natural philosophy too were doomed to give way to something which should immortalise Davy, and receive, itself, a strong impress from his mind—we allude, of course, to chemistry, the study of which he began in November or December of this year, when he was just nineteen.

"Several incidents, which will be mentioned hereafter, enable me to fix the time with precision. He appears to have entered on this study merely as a branch of his professional knowledge, and to have followed it at first chiefly theoretically. His early chemical reading was confined to two works of a very different description,—'Lavoisier's Elements of Chemistry,' and 'Nicholson's Dictionary of Chemistry,'—the one distinguished for its admirable logic, precision of reasoning, and boldness of speculation compared with any that had preceded it; the other an indifferent collection of facts and opinions, of various times and merit,—a kind of border tract between the old and new doctrines, and hardly worthy of the name of the author it bore. This new study seems very soon to have excited in his mind a most lively interest. He was not satisfied with merely reading, and acquiring the ideas of others; he criticised the theoretical views of the great French philosopher; doubted, rejected, and advanced speculations of his own. And speculation appears to have led him to experiment, and experiment to further speculation, with such rapid progress, that in a few months he formed a new hypothesis, and flattered himself that he had triumphed over an important part of the doctrine of the French school." 33.

His experiments were limited by his means, and not more extensive than those with which Priestly and Scheele began. His apparatus would appear to have consisted chiefly of phials, wine-glasses, and tea-cups, tobacco-pipes, and earthen crucibles; and his materials were chiefly the mineral acids and the alkalies, and some other articles which are in common use in medicine. He began his experimental trials in his bed-room. Here there was no fire, and when he required it, he was obliged to come down to the kitchen with his crucible. His experiments were at first simple, and valuable only from making him familiar with his tools, and nourishing his enthusiasm for his science. The rapidity, says his biographer, with which he advanced in his new pursuit is strongly indicated by the circumstance that, in the April following, in the short space of four months, he was in correspondence with Dr. Beddoes, relative to his researches on "Heat and Light," and a new hypothesis on their nature, to which Dr. Beddoes became a convert. The results of these researches were the chief subject of his first publication, "Essays on Heat and Light," &c., which appeared in 1799, and were in part written a few months after he had commenced the study of chemistry. For he carried to that all the powers of a strong mind, and the force of an ardent enthusiasm. And he became acquainted with Mr. Gregory Watt, son of the elder Watt, and with Mr. Davies Gilbert. With them he examined the rich mines, and the produce of the land and sea. If he did not study medicine in the right way he practised it; for—

"It would appear that he applied himself with earnest zeal to his professional studies and duties, and that he gained equally the good opinion of Mr. Borlase and of his patients, especially of the poorer class, to whom he showed particular kindness. Mr. Borlase's high opinion of him, and estimation of his deserts, was proved by an act of generous kindness, which will be noticed in its place; and this gentleman's sister, Mrs. Foxell, bears testimony to the humane way in which he behaved towards those in humbler life. She has told my sister, that, 'in all cases of distress, she used to call upon him, and always found him ready to render every assistance in his power; and that she had often heard him say that he wished to make himself useful to his fellow-creatures.'" 41.

He read Shakspere, and Milton, and Gibbon, fished, shot snipes, and winged woodcocks, and was altogether a very happy fellow, more happy, we dare say, than at many a soirée of the Royal Society, when aristocratic acquaintances had dammed up in some sort the impulses of youth and nature. He writes in one of his note-books:—"I have neither riches, nor power, nor birth to recommend me; yet, if I live, I trust I shall not be of less service to mankind and to my friends than if I had been born with these advantages." And his mind is portrayed in a fragment which he left, representing, no doubt, his actual feelings:—"I gradually became conscious of my powers, by comparing them with those of others. That solitary enthusiasm, however, which constituted my independence was never lost. I was no longer anxious to know what others thought of me, and I panted little after the breath of fame. Hence, agitated by no passion but the love of truth, the desire to see things in their real light counteracted every other desire. My conversations were plain and simple; I perceived that circumstances and the development of my moral powers had produced, or, rather, gradually unfolded, a new moral character. It was this character that I sought to improve, by casting from me every trait of hypocrisy and concealment. I considered all my possible relations with men, and I found no one which could again possibly turn me over to dependence." These early aspirations were hardly, we fear, realized.

Soon after his correspondence with Dr. Beddoes began, the latter offered him the situation of superintendent of the Pneumatic Institution, which had been established at Clifton for the purpose of trying the medicinal effects of different gases. After some negociation respecting the terms of the appointment (in which Mr. Davies Gilbert aided him in a very friendly manner), he gladly accepted it, with the consent of all his friends, excepting Mr. John Tonkin (who had hoped he would have settled at Penzance), and with the approval of Mr. Borlase, who (the period of his apprenticeship not being expired), in a very generous manner, released him from "all engagements whatever, on account of his excellent behaviour;" adding, "because, being a youth of great promise, I would not obstruct his present pursuits, which are likely to promote his fortune and his fame." And on the 2d of October, 1798, he left his home, to enter upon his public career, before he was twenty years old.

Everybody has heard of Dr. Beddoes, and of his pneumatic institution. Few have seen him, and many, we dare say, would like a view of his portrait, painted by *young* Humphry and *Sir* Humphry Davy. Thus young Humphry, just settled at Clifton, limns him and his wife.

"Now for the inhabitants, and first, Dr. Beddoes, who, between you and me, is one of the most original men I ever saw—uncommonly short and fat, with little elegance of manners, and nothing characteristic *externally* of genius or science; extremely silent, and in a few words, a very bad companion. His behaviour to me, however, has been particularly handsome. He has paid me the highest compliments on my discoveries, and has, in fact, become a convert to my theory, which I little expected. He has given up to me the whole of the business of the Pneumatic Hospital, and has sent to the editor of the Monthly Magazine a letter to be published in November, in which I have the honour to be mentioned in the highest terms. Mrs. Beddoes is the reverse of Dr. Beddoes—extremely cheerful, gay, and witty; she is one of the most pleasing women I ever met with. With a cultivated understanding, and an excellent heart, she combines an uncommon simplicity of manners. We are already very great friends. She has taken me to see all the fine scenery about Clifton; for the Doctor, from his occupations and his bulk, is unable to walk much." 46.

And thus Sir Humphry, just about to shuffle off his own mortal coil, paints the likeness of an old friend :—

"Beddoes was reserved in manner, and almost dry; but his countenance was very agreeable. *He was cold* in conversation, and apparently much occupied with his own peculiar views and theories. Nothing could be a stronger contrast to his apparent coldness in discussion, than his wild and active imagination, which was as poetical as Darwin's. He was little enlightened by experiment, and, I may say, little attentive to it. He had great talents, and much reading, but had lived too little amongst superior men. On his death-bed he wrote me a most affecting letter, regretting his scientific aberrations. I remember one expression: 'Like one who was scattered abroad the *avena fatua* of knowledge, from which neither branch, nor blossom, nor fruit has resulted, I require the consolations of a friend.' Beddoes had talents which would have exalted him to the pinnacle of philosophical eminence, if they had been applied with discretion." 49.

How harsh, and how unjust was that *mot* upon Beddoes—"he was the discoverer of one thing, for he was the discoverer of Davy." There was good society at Clifton at that time, something more than the old women who, at present, seem to form nine-tenths of the inhabitants. There met then at Beddoes' house, Southey, and Coleridge, and Tobin, and the late Mr. Poole. Young Davy was among kindred spirits.

In the beginning of 1799, writes his biographer, he took up his residence in a house in Dowery-square, Clifton, which was provided with a laboratory, and fitted up for the purposes of the institution. Here he applied himself with the greatest assiduity and zeal to various research;—as to the completing his experiments for, and finishing his essays on Heat and Light, before alluded to, which were published this year; the determining of the nature of the epidermis of the hollow plants,—an investigation he undertook in consequence of the accidental observation of a child;—but, above all, investigating the effects of the gases in respiration. Of these, the nitrous oxide was one of the first he experimented upon. Its agency he found to be of a very novel and wonderful kind, contrary to all expectation, and almost exceeding belief. The discovery gave a particular direction to his inquiries, and may be considered as the origin of that work which contained their results, and which established his character as a chemical philosopher. It was published in the Summer of 1800. Even before it was out of the press, he had embraced another train of research, new and most important;—he had begun that series of galvanic experiments which ultimately led to some of his greatest discoveries.

Dr. Davy adds, and his general observations are characterised by much truth :—

"In regard to the formation of his scientific character, this was a very critical period of his life; and it is well shown in the manner in which he treated the different subjects of inquiry to which I have briefly alluded. He started in his career of chemical inquiry, as he had before done in metaphysical, in all the presumption of youthful daring, with undue confidence in his speculations, and in speculation in general. His first published essays on Heat and Light are very characteristic, as the bold attempts of an original and enterprising mind, and bearing the stamp, at the same time, of youth and of genius,—in the faults belonging to the one, and the redeeming qualities of the other, marked, as they are by invention, by a certain strength and precision of reasoning power, and by much ingenuity both of ideas and experiments, and also by want of caution, and the precipitancy of a very young man, which sometimes appears as a defect of modesty. He himself, with surprising rapidity, detected the errors into which he had fallen; and he did so probably mainly in consequence of the other researches in which he immediately engaged, of a description fitted to afford precise results—and yet such that no ingenuity *à priori* could have conceived, and some of them so singular and surprising, that no imagination could have invented. He must now have become acquainted practically with the value of the Baconian maxim,—that first aphorism of the 'Novum Organum Scientiarum,'—'Homo Naturæ minister, et interpres, tantum facit et intelligit quantum de Naturæ ordine re vel mente observaverit: nec amplius sit, aut potest.' Indeed I am disposed to think, that after the publication of these essays, he almost immediately acquired, and for a short time felt, even an undue aversion to hypothesis. His own opinions expressed respecting these essays are strongly indicative of his altered views. 'I began the pursuit of chemistry by speculations and theories: more mature reflection convinced me of my errors, and the limitation of our powers, the dangers of false generalization, and the difficulty of forming true ones.' This I find written in darker ink between the lines of a note-book kept in 1799, consisting chiefly of speculative views concerning the connexion of life and chemical action, or of physiology and chemistry. In another place, bearing date of August of the same year in which his essays appeared, is the following reflection:—'When I consider the variety of theories that may be formed on the slender foundation of one or two facts, I am convinced that it is the business of the true philosopher to avoid them altogether. It is more laborious to accumulate facts than to reason concerning them; but one good experiment is of more value than the ingenuity of a brain like Newton's.' And, in the same note-book, written about the same time as the preceding, alluding to his essays, he says, 'I was perhaps wrong in publishing with such haste a new theory of chemistry. My mind was ardent and enthusiastic. I believed that I had discovered the truth. Since that time my knowledge of facts is increased,—since that time I have become more sceptical.'" 55.

All this is indicative of a strong mind. A weaker one would have gone on floundering in hypothesis, and bewildered in the maze of words and mysticism, that form the tangled web of transcendental philosophy. Davy, however, perceived the utter futility of this, although it is evident, from detached papers, and from his last works, that his early predilection for metaphysical abstractions, and speculations de omnibus rebus et quibusdam aliis, was only mastered by an effort, put off when he put on his working dress, and worn with his morning gown and slippers, perhaps with his pumps and silk stockings. However this may be, it is certain that he saw the true road to chemical, as to all other discoveries in the natural sciences, namely, strict analysis and stern induction. This was the secret of Davy's success, and one would have thought there was now-a-days no need of defending this. Yet opinions make their revolutions like the seasons, and few have been so completely overthrown as not to rise again in their turn. Such would seem to be the case with the ancient synthetical method. It has revived, or threatens to revive, in Germany, and its fruits may be looked for in a goodly crop of unintelligible theories. But Davy, as a working man, took a different view, and resolved to analyse and to observe strictly. "The perfection," he thought, (for he noted what he thought) "of chemical philosophy, or the laws of corpuscular motion, must depend on the knowledge of all the simple substances, their mutual attractions, and the ratio in which these attractions increase or diminish, with increase or diminution of temperature. These being ascertained, chemistry would become a science so far generalised as to enable calculations to be formed with regard to the result of any new apposition of particles. The first step towards these laws will be the decomposition of those bodies which are at present undecompounded." The bodies alluded to were the fluoric, muriatic, and boracic acids, the alkalies, and earths; the elements of all which, in a few years from this time, he succeeded in developing: and even at this time he attempted it in regard to the acids. But the attempts were failures, partly no doubt from his inexperience, partly from the insufficiency of means.

"The extraordinary zeal with which he devoted himself to research at this time, and his great powers of application, are forcibly shown in the rapidity of his labours. His 'Researches' were published in the summer of 1800; a work so laborious might have sufficed for the life of an ordinary man; the materials of it were collected and put together in an almost incredibly short time. In a note-book now before me, there is a rough draft of a preface, in which he says, 'These experiments have been made since April, 1799, the period when I first breathed nitrous oxide. Ten months of incessant labour were employed in making them; three months in detailing them.' So intense indeed was his application, and so little his regard for health or even life, which was often endangered, and once nearly lost from the breathing of carburetted hydrogen, that he was under the necessity of leaving the laboratory for a time, when most anxious to complete his inquiries, and seek the renewal of health and strength in his native air." 58.

He remained at Penzance a month, fished, shot, rambled, and wrote poetry as usual, and took with him a case of chemical apparatus considerably larger than the one which, some years after, he carried with him on his continental travels. He returned in due time to Bristol, and wrote all sorts of things, or, rather, he resolved to write them, for many were begun, and none, so far as we can see, were ended. It would be hard indeed, if they were.

From the thousand and one scraps produced by him, and preserved in part by his biographer, we shall only quote his theory of dreams.

"Our waking existence is composed of impressions, ideas, and feelings, of different degrees of vividness, which succeed each other in what may be called trains. The past is simple memory, the future is analogy, and the present is made up of impressions, sometimes, though rarely, simple, but often mixed or associated with the past and the future. In dreams, all the ideas occurring seem to be of one degree of vividness. There is neither past nor future, no mixture of impressions with ideas; the feelings occur as in waking, though seldom connected with what may be called the secondary reflective feelings, those produced when an action, at first pleasant, after a time becomes the contrary. The mixtures of memories and analogies, which are mingled, in waking, by the peculiarly modifying and connecting impressions in the order of nature, are here called up and modified only by slight organic feelings; generally so slight as to lose their independent existence, and can only be traced by impressions which are connected with them." 63.

This theory is itself somewhat dreamy. If any body understands any more of dreams, on reading it, than he did before, he must understand it better than we do. Amongst other things, Davy sketched the programme, and executed fragments of a poem in blank verse, named "Moses," the the subject being the deliverance of the Israelites from Egypt. Some of the passages are really rich in poetic beauty. Take one:—

"And loud she struck the harp, and raised the song,
Her ebon tresses waving in the wind;
Her dark eye sparkling, and her bosom
Throbbing with transport high.—'Thou, thou art he,
The chosen one of God,—the man foretold
The saviour of thy people. Prophet chief,
And lawgiver of Israel! at thy birth
Deliver'd to the waters, yet preserved,
By hand unhallow'd,—from the royal pomp
Of Pharaoh, and the dark idolatry
Of Egypt's kingdom led to know thy God,
In nature and in solitude to feel
His mighty inspirations! Go then forth:
In all the high unbroken strength of hope
Proclaim the Eternal One,—declare his will.
Let Egypt and the kindred nations know
That *He alone* is God!—that He will free
In terror and in wrath his chosen seed,
Exalt the oppressed, tread the tyrant low,
And scatter as the sand upon the blast
The people that rebel against His will.
Go forth his servant,—go deliverer!"

An epoch in his life was approaching, for, at the recommendation of Dr. Hope of Edinburgh, Count Rumford applied to him to fill a situation at the Royal Institution. In a letter to his mother, he writes:—"You have, perhaps heard of the Royal Philosophical Institution, established by Count Rumford, and others of the aristocracy. It is a very splendid establishment, and wants only a combination of talents to render it eminently useful. Count Rumford has made proposals to me to settle myself there, with the present appointment of assistant lecturer on chemistry, and experimenter to the Institute; but this only to prepare the way for my being in a short time sole professor of chemistry, &c.; an appointment as honourable as any scientific appointment in the kingdom, with an income of at least £500 a-year."

In March, 1801, the engagement was actually concluded, and Davy was settled in London. At first he was only assistant lecturer, and director of the laboratory, but on the 31st of May, 1802, he was formally appointed Professor of Chemistry.

In the Spring of 1801, he delivered his first lecture. It appears to have excited a sensation, which his subsequent ones kept up. The following accounts of them by auditors will be read with some degree of interest.

"Sir Joseph Banks, Count Rumford, and other distinguished philosophers, were present. The audience were highly gratified, and testified their satisfaction by general applause. Mr. Davy, who appears to be very young, acquitted himself admirably well. From the sparkling intelligence of his eye, his animated manner, and the *tout ensemble*, we have no doubt of his attaining distinguished excellence.

'The sensation created by his first course of lectures at the institution,' says Mr. Purkis, 'and the enthusiastic admiration which they obtained, is at this period hardly to be imagined. Men of the first rank and talent,—the literary and the scientific, the practical and the theoretical,—blue-stockings and women of fashion, the old and the young, all crowded, eagerly crowded the lecture-room. His youth, his simplicity, his natural eloquence, his chemical knowledge, his happy illustrations and well-conducted experiments, excited universal attention and unbounded applause. Compliments, invitations and presents were showered upon him in abundance from all quarters; his society was courted by all, and all appeared proud of his acquaintance.'" 80.

If Davy *was* spoilt, who could wonder at it? For a man of his ardent temperament, he was in as dangerous a situation as could be. However, he succeeded as a lecturer completely. He was made of the stuff to do so. He had enthusiasm, that was the great point. Nobody ever did, and nobody ever will move others, who remains unmoved himself. A man who does not feel burlesques feeling, and for one to appeal to our passions who evinces none, is to put a sort of affront upon us, or, rather, to tell us to laugh or to cry, at the very instant that his own example shews the absurdity of either. Enthusiasm even redeems extravagance, and, awakening sympathy, it throws a veil over defects.

It must not be supposed that good lecturers are ready made, or that there are heaven-born popular speakers any more than there are heaven-born shoemakers or tailors. Eloquence, if partly a gift, is fully as much an acquirement. We will not go back to Demosthenes or Cicero, who worked, like cart-horses, at their craft, but content ourselves, with our actual hero, Mr. Davy. His brother observes :—

"I shall make a few remarks, conveying the impression which they have left on my mind, and recording the manner in which they were conducted. He was always in earnest; and when he amused most, amusement appeared most foreign to his object. His great and first object was to instruct, and, in conjunction with this, maintain the importance and dignity of science; indeed, the latter, and the kindling of taste for scientific pursuits, might rather be considered his main object, and the conveying instruction a secondary one. His lectures were almost invariably written expressly for the occasion, not a repetition of lectures; so that the same audience, year after year, might attend, without being wearied. He commonly wrote his lecture the day before he delivered it. On this day he generally dined in his own room, and made a light meal on fish. He was always master of his subject; and composed with great rapidity, and with a security of his powers never failing him. Latterly, he trusted a good deal to notes; and, excepting on particular occasions, wrote little more than the parts which he wished to make most impressive, especially the beginning and termination. It was almost an invariable rule with him, the evening before, to rehearse his lecture in the presence of his assistants, the preparations having been made and everything in readiness for the experiments; and this he did, not only with a view to the success of the experiments, and the dexterity of his assistants, but also in regard to his own discourse, the effect of which, he knew, depended upon the manner in which it was delivered. He used, I remember, at this recital, to mark the words which required emphasis, and study the effect of intonation; often repeating a passage two or three different times, to witness the difference of effect of variations in the voice. His manner was perfectly natural, animated, and energetic, but not in the least theatrical. In speaking, he never seemed to consider himself an object of attention; he spoke as if devoted to his subject, and as if his audience were equally devoted to it, and their interest concentrated in it. The impressiveness of his oratory was one of its great charms; in this he consulted only his own taste and feelings; but at the same time, by a considerable portion of his auditors, it must have been received as a compliment to their own taste and feelings, and powers of understanding; and, in giving them credit for acquirements, no doubt many flattered themselves they possessed them, or had a desire excited to attain them. His experiments were devised on the same principle, not of amusing and pleasing, but of illustrating his discourse, and demonstrating either important properties of bodies, or principles of the chemical action of bodies: he took great care that they should not appear to have been introduced for show, and to excite merely wonder, even when most brilliant and wonderful. And his eloquence,—the declamation, as it might be called by some, in which he indulged on the beauty and order of nature, in which he ascended from the works to the great Artificer, and from the admirable design every where apparent to the infinite wisdom of the Author of the universe,—his eloquence, I believe, on these topics was so well received, because it was not affected; merely his own strong impressions and feelings embodied in words, and delivered with an earnestness which marked their sincerity." 93.

Here we see the "eloquent" lecturer practising tolerably hard—dining on fish—writing his lecture—getting the *hits* by heart—rehearsing the performance—studying tones—marking emphasis—going to work, in short, as systematically with a piece of declamation as he would with the preparation of potassium. And he was quite right; for good lectures or good speeches are like all other good things, they are not manufactured without trouble. If we heard the orator in his private room, how much it would mar our opinion of the oration.

We have seen Davy in the lecture-theatre, let us turn to him in the laboratory.

"In the laboratory, where my brother spent a great portion of every day that he was in town and at leisure, he was unremittingly engaged in original experiments; and even in his absence the operations were not suspended, they were continued by his assistants, according to the directions which he had given; and when he returned, he finished the experiment, or examined the results. Nothing was left to memory; an entry was made in a large book, kept for the purpose, of all that occurred, written either by himself, or by an assistant from his dictation; not, indeed, in minute detail, for that would have occupied too much time, but briefly, for aiding the memory, and minutely only in regard to weight and measure, and what was most important and characteristic. In his inquiries there never was any mystery or concealment, but the most perfect openness. The register of experiments was left open; he received his friends in the laboratory, and conversed with them on the objects of inquiry in progress; and however intensely engaged, he was always accessible. I can never forget his manner when occupied in his favourite pursuit; his zeal amounted to enthusiasm, which he more or less imparted to those around him. With cheerful voice and countenance, and a hand as ready to manipulate as his mind was quick to contrive, he was indefatigable in his exertions. He was delighted with success, but not discouraged by failure; and he bore failures and accidents in experiments with a patience and forbearance, even when owing to the awkwardness of assistants, which could hardly have been expected from a person of his ardent temperament. And his boldness in experimenting was very remarkable: in the operations of the laboratory danger was very much forgotten, and exposure to danger was an every-day occurrence. Considering the risks run, and the few, if any, precautions taken against accidents, it is surprising how small a number of injuries were received. The only two serious wounds that I recollect he sustained, were in the hand and eye; the one, from receiving on his hand a quantity of melted potash; the other, from an explosion of a detonating compound. Had his constitution been bad, the use both of hand and eye would probably have been impaired; indeed, the eye ever after retained the mark of the wound inflicted on the transparent cornea, and never perfectly recovered its strength." 96.

This is just what might be expected. It would hardly be supposed that in his private habits he was very systematic. Nor was he.

"Of my brother's mode of living, and of some of his habits whilst he was at the Royal Institution, I shall also speak from my own knowledge. As long as he was a bachelor, he was perfectly satisfied with his rooms at the Institution, in which he considered chiefly utility, and thought little of comfort, and much less of luxury. He showed great carelessness in all that related to their furniture and appearance. These were to him matter of indifference. I believe the furniture was merely what belonged to them when he first took possession, and that he made no addition to it or alteration. The only thing elegant that I recollect in his sitting-room was an ornamental little porcelain Venus, which was a present to him from his early friend, Mr. Wedgewood; it was of his manufacture, and an admirable specimen of art. Letters and papers he very seldom arranged, and his rooms were commonly littered with them. Occasionally they were collected and thrown together in a large cupboard. I remember once his commissioning me to look over this great collection, and to burn such as appeared of no interest. Amongst them were very many letters of the highest compliment, and some of kind advice from anonymous writers, or declared friends, pointing out, on his commencing lecturing at the Institution, what was considered faulty in his manner, and even in his pronunciation. But they were most commonly of a laudatory kind; and of this kind were several copies of verses, written in female hands, showing that he had excited no ordinary interest in their breasts, and that their admiration was of a very exalted kind." 97.

"His way of living during this period was of the most easy kind. Except when preparing a lecture, as already mentioned, he seldom dined in his apartments at the Institution: his invitations to dinner amongst his friends were so numerous that he was, or might have been, constantly engaged; and after dinner he was much in the habit of attending evening parties, devoting the evening to amusement; so that, to the mere frequenters of such parties, he must have appeared a votary of fashion rather than of science. When his pursuits did not keep him in town, he often made short visits to friends residing in the neighbourhood of London, or went to some trout stream, of which there are so many good ones within twenty or thirty miles of the metropolis, and breathed the fresh air by the river side, and enjoyed the country and his favourite exercise and amusement of fishing together.

And during the vacations, when he had some months' leisure, he commonly made longer excursions. At different times he explored most parts of Great Britain, including even the distant parts of Scotland, and the Hebrides; and more than once he travelled through Ireland. His object in these journeys, was partly amusement and the acquiring of general information, and partly the study of the geological structure of the kingdom, and the phenomena of rock-formations in relation to geology as a science, and partly the collecting of agricultural knowledge. He made sketches of remarkable features of rock-scenery, and of peculiarities in their appearance; and he collected specimens of rocks, and minerals, and soils, which were deposited on his return in the museum of the Institution, or in its laboratory for examination. When he lectured either on the subject of geology or agriculture, he availed himself of them, and of paintings made from his sketches, which were not less serviceable in illustrating his descriptions and doctrines than the specimens of rocks and minerals themselves. His power of extracting information was great; it was constantly employed in these excursions, and I believe gave a strong idea of talent and capacity to comparatively uninstructed men." 99.

That Davy, at this time, was happy, may be well conceived. His brother assures us that he was eminently so. Dr. Davy resided at the Institution between 1808 and 1811, and their bed-rooms were only separated by a wainscoat partition. In going to bed, and rising, and sometimes in the dead of night, Dr. Davy used to hear him, in a loud voice, reciting favourite passages in prose or verse, or declaiming some composition of his own, or humming some angler's song. What a bore the lecturer must have been. Yet happy as he was, perhaps, Davy was now laying the foundation of much future chagrin. He was becoming a sort of man of fashion, and was trying to reconcile the aristocratic trifler with the man of science. An impossible task.

He became, at this time, a philosophical tanner, and an agricultural chemist; but it was in electro-chemical science that he engaged heart and hand. In 1801, the Voltaic pile was invented, and water was decomposed by it. Davy's mind was strongly impressed by the discovery, and his experiments with this new and grand agent of solving the mysteries of the natural world were at once begun, and ever after pursued. No great results were obtained at first, (how seldom they are,) but, in 1806, success opened to him. Potassium was obtained on the 6th of October of that year, and sodium a few days after.

" The extreme delight which he felt, when he first saw the metallic basis of potash, can only be conceived by those who are familiar with the operations of the laboratory, and the exciting nature of original research; who can enter into his previous views, and the analogies by which he was guided, and can comprehend the vast importance of the discovery, in its various relations to chemical doctrine; and, perhaps, not least, who can appreciate the workings of a young mind with an avidity for knowledge and glory commensurate. I have been told by Mr. Edmund Davy, his relation and then assistant, now professor of chemistry to the Dublin Society, that when he saw the minute globules of potassium burst through the crust of potash, and take fire as they entered the atmosphere, he could not contain his joy—he actually bounded about the room in extatic delight: and that some little time was required for him to compose himself sufficiently to continue the experiment.

Never, perhaps, was a chemical investigation more intensely interesting than the one under consideration; and never, perhaps, in so short a time were so many new and surprising facts developed. The Bakerian Lecture in which they are described attests this most fully; it occupies forty-four quarto pages, and almost every page contains a new result. Notwithstanding it was a first sketch, and relating to phenomena altogether new and marvellous, it scarcely required any after correction, excepting in that part which treats of the volatile alkali; and though it was written on the spur of the occasion, before the excitement of the mind had subsided, yet it bears proof only of the maturest judgment; the greater part of it is as remarkable for experimental accuracy as for logical precision. This is the more worthy of notice, as when he composed it he was in a feverish state,—the prelude to a severe attack of illness, which was very near proving fatal,—and his great apprehension was, that he should die before he had published his discoveries; in consequence of which dread, he applied himself the more unremittingly to the task of detailing them." 110.

The upshot of all this excitement was a fever, which had well nigh killed him, and kept him in his bed nine weeks. His biographer states some circumstances which prove the interest felt for him. Had he been of the highest rank in society, says Dr. Davy, greater attentions could not have been paid him, more anxious inquiries could not have been made after him. When he was at the worst, his physicians reported his state concisely in writing, for the information of the many who called to ask. His physicians attended him with the greatest assiduity, and in the most friendly and disinterested manner. Two of them, Dr. Babington and Dr. Frank, were previously his friends; Dr. Baillie, who was called in when his illness was most threatening, was not behind them in kindness, disinterestedness, and attention. Dr. Davy mentions, in a note, a fact quite worthy of Dr. Baillie, and consistent with his character.

"The disinterestedness of this eminent physician, a quality for which he was so much distinguished, was marked on this occasion by his returning to my brother (as he himself informed me) a fee of fifty pounds, accompanied by a very friendly note, to the intent that he could not act otherwise towards a man of science." 111.

We need not detail Davy's researches on muriatic acid, or his demonstration of the properties of chlorine, &c. Our business is mainly with his personal history. In 1803, he was elected a Fellow, and in January 1807, one of the Secretaries of the Royal Society,—associated in office with those distinguished men, Dr. Hyde Wollaston and Dr. Thomas Young;—the latter in the capacity of Foreign Secretary—the former of Senior Secretary. He held the appointment, annually re-elected, until 1812,—when he resigned it,—shortly after retiring from the Royal Institution, on his marriage. It was a situation peculiarly agreeable to him; and the attainment of it, he justly valued as a great honour. But Davy had an idea of turning physician, and resuming his interrupted professional studies. A fashionable physician, or a chemical one, we have no doubt that he would have made —a good practical physician, we are pretty certain that he would not. However, he entered his name at Cambridge, and kept some terms there. He was probably induced to form this plan by a prospect of fortune, in a professional career, infinitely greater than he had any right to expect from the mere prosecution of science. From the latter he derived a competency, and little more; from the other, he might calculate on making an independence, and on acquiring means of his own for prosecuting his favourite pursuits. He wisely abandoned the scheme. And, perhaps as wisely, he had previously declined an invitation to enter the church. Some of his powerful friends, especially the Bishop of Durham and Sir Thomas Bernard, were desirous he should do so, with the persuasion that his eloquence might be of efficient service in the cause of religion, and holding out to him the brightest prospects of preferment. He contented himself with giving his aid to the cause in connexion with science.

During the period he continued to lecture in the Theatre of the Royal

Institution, his popularity was constantly increasing, and the size of his audience kept pace with it. Latterly it was scarcely less than 1000. His lectures were so attractive, that had he chosen to have retired from the Institution, like his predecessor Dr. Garnet, and to have opened a course on his own account, he might probably have acquired a large income. In consequence of his reputation and discoveries he was twice invited to deliver courses of lectures to the Dublin Society successively, in 1810 and 1811; and each time he was received not merely in a distinguished, but in an enthusiastic manner. On each occasion resolutions were passed on the part of the Society of a very flattering kind, expressive of thanks and gratification, and the compliment was crowned by Trinity College, Dublin, conferring on him the degree of Doctor of Civil Law.

Among the experiments which Davy prosecuted about this time, there was one of great interest and no slight hazard, which philosophers often conduct to no better result, sometimes indeed to a worse, than other people. This was the experiment of matrimony. The man of enthusiasm may well be supposed to have gone about this with ardour. Mrs. Appreece was the lady whom Davy selected, and he seems to have been such a lover as a lady loves. He writes in this strain :—

"My dear Mother,—You possibly may have heard reports of my intended marriage. Till within the last few days it was mere report. It is, I trust, now a settled arrangement. I am the happiest of men, in the hope of a union with a woman equally distinguished for virtues, talents, and accomplishments.

"You, I am sure, will sympathise in my happiness. I believe I should never have married, but for this charming woman, whose views and whose tastes coincide with my own, and who is eminently qualified to promote my best efforts and objects in life." 133.

And he breathes his hopes and fears, and transports, into his brother's ear in this fashion :—

"My dear John,—Many thanks for your last letter. I have been very miserable. The lady whom I love best of any human beings has been very ill. She is now well, and I am happy. Mrs. Appreece has consented to marry me; and when the event takes place I shall not envy kings, princes, or potentates." 134.

The Prince Regent made him a marriage present in the shape of a knighthood, which Davy was not a little proud of. He married accordingly, and seems to have had reason, in every way, pecuniary as well as domestic, to be contented with his bargain. He was appointed Honorary Professor to the Institution, published his Elements of Chemical Philosophy soon after his marriage, and dedicated it to Lady Davy. He published too his "Agricultural Lectures," for the copyright of which he got 1000 guineas, and 50 guineas for each edition, as he himself says, a "fair" price. Amongst a number of quotations from Davy's note-book we select one, which contains, in petto, his opinions on the future prospects of this country. "Sooner or later," he says, "our colonial empire must fall in due time, when it has answered its ends. The wealth of our island must be diminished, but the strength of mind of the people cannot easily pass away; and our literature, our science, our arts, and the dignity of our nature, depend little upon external relations. When we had fewer colonies than Genoa, we had Bacons and Shakspeares. 'The wealth and prosperity of the country are only the *comeliness* of the body, the fulness of the flesh and fat; but the spirit is independent of them: it requires only muscle, bone, and nerve, for the true exercise of its functions. We cannot lose our liberty, because we cannot cease to *think*; and ten millions of people are not easily annihilated.' " Without pinning our faith to Davy as a politician, we think we may very safely coincide in this last opinion. Millions of Englishmen can neither be exterminated, nor mentally gagged.

While making a detonating compound of chlorine and azote, he received a severe wound in the cornea, from which he did not recover for four or five months. In the Autumn of 1813, he obtained permission from the French Government, on account of his scientific eminence, to pay a visit to the Continent, and, on the 15th of October, he crossed the Channel, accompanied by Mr. Faraday, "as his assistant in experiments and writing." He went direct to Paris, where he remained two months, and assisted in determining the nature of iodine. In his last illness, he beguiled the hours of gloom, by sketching reminiscences of some of the great men of science that he met. These are the light touches that always charm, and we shall transfer some to our pages.

" *Vauquelin* was in the decline of life when I first saw him in 1813,—a man who gave me the idea of the French chemists of another age; belonging rather to the pharmaceutical laboratory than to the philosophical one: yet he lived in the Jardin du Roi. Nothing could be more singular than his manners, his life, and his ménage. Two old maiden ladies, the Mademoiselles de Fourcroy, sisters of the professor of that name, kept his house. I remember the first time that I entered it, I was ushered into a sort of bed-chamber, which likewise served as a drawing-room. One of these ladies was in bed, but employed in preparations for the kitchen; and was actually paring truffles. Vauquelin wished some immediately to be dressed for my breakfast, and I had some difficulty to prevent it. Nothing could be more extraordinary than the simplicity of his conversation;—he had not the slightest tact, and, even in the presence of young ladies, talked of subjects which, since the paradisaical times, never have been the objects of common conversation." 166.

" *Cuvier* had even in his address and manner the character of a superior man;—much general power and eloquence in conversation, and a great variety of information on scientific as well as popular subjects. I should say of him, that he is the most distinguished man of *talents* I have known; but I doubt if he is entitled to the appellation of a man of genius.

" *De Humboldt* was one of the most agreeable men I have ever known; social, modest, full of intelligence, with facilities of every kind: almost *too fluent* in conversation. His travels display his spirit of enterprise. His works are monuments of the variety of his knowledge and resources."

" *Gay Lussac* was quick, lively, ingenious, and profound, with great activity of mind, and great facility of manipulation. I should place him at the head of the living chemists of France." 167.

" *La Place*, when a minister of Napoleon, was rather formal and grand in manner, with an air of protection rather than of courtesy. He spoke like a man not merely feeling his own power, but wishing that others should be immediately conscious of it. I have heard, from good authority, that he was exceedingly proud of his orders, and that he had the star of the order of Re-union affixed to his dressing-gown. This was in 1813. In 1820, when I saw him again, his master had fallen. His manners were altered. He was become mild and gentlemanlike; and had a softer tone of voice, and more grace in the forms of salutation. I remember the first day I saw him, which was, I believe, in November, 1813. On my speaking to him of the atomic theory in chemistry, and expressing my belief that the science would ultimately be referred to mathematical laws, similar to those which he had so profoundly and successfully established with respect to the mechanical properties of matter, he treated my idea in a tone bordering on contempt, as if angry that any results in chemistry could, even in their future possibilities, be compared with his own labours. When I dined with him, in 1820, he discussed the same opinion with acumen and candour, and allowed all the merit of John Dalton. It is true our positions had changed. *He* was now amongst the old aristocracy of France, and was no longer the intellectual head of the new aristocracy; and, from a young and humble aspirant to chemical glory, I was about to be called, by the voice of my colleagues, to a chair which had been honoured by the last days of Newton." 168.

From Paris he proceeded to Fontainbleau, where he semi-prophecied the fall of Napoleon, and thence he went by Genoa and Florence to Rome, observing in most places, experimenting in many, and writing poetry in all. At Milan he saw Voltá, whom he draws:—" Voltá I saw at Milan, in 1814, at that time advanced in years, I think nearly 70, and in bad health. His conversation was not brilliant; his views rather limited, but marking great ingenuity. His manners were perfectly simple. He had not the air of a courtier, or even of a man who had seen the world. Indeed, I can say generally of the Italian savans, that, though none of them had much dignity or grace of manner, yet they were all free from affectation."

After his return from the Continent, in 1814, he entered on the investigation of " fire-damp," and by a highly beautiful series of inductions and experiments he arrived at the construction of the safety-lamp. A sufficient answer to the sneer, if any are fools enough now to venture on it, at *feelosofy*, and *feelosofers*, and to the question " what good comes of abstract science?" Well might Mr. Playfair quote the sarcasm of Bacon:—" Jam per tot annorum spatia, vix unum experimentum adduci potest quod ad hominum statum levandum et juvandum spectat, et philosophiæ speculationibus ac dogmatibus, neve acceptum referri possit;" and add:—" This is exactly such a case as we should choose to place before Bacon, were he to revisit the earth, in order to give him, in a small compass, an idea of the advancement which philosophy has made, since the time when he had pointed out to her the route which she ought to pursue."

Nobody who knows any thing of governments would expect that they would substantially reward the mere saviour of lives. That would be robbing the political pimps and whippers in, for whom the " nation" reserves its pensions. A baronetcy, indeed, being cheap, and depriving no voter or any dealer in votes of his due, might be conferred, and was so. The public is *left* on these occasions to pay its benefactor, the same public being *made* to pay the factions that rough-ride it. The coal-owners, therefore, were allowed to be as generous as they chose to Sir Humphry. He received letters of thanks from various individuals, and from the united colliers of Whitehaven; a vote of thanks from the coal trade of the North of England, —from the grand jury of Durham,—and from the chamber of commerce at Mons. And besides thanks, as a permanent mark of their obligations, he was presented with a service of plate of the value of £2,500, at a public dinner given to him at Newcastle on the 11th October, 1817, at which Mr.

Lambton, now Earl of Durham, presided. He received also a splendid silver-gilt vase from the late Emperor Alexander of Russia, accompanied by a letter from the Emperor himself, expressive of his sentiments in relation to his important discovery. We cannot refrain from quoting a conversation, reported by Mr. Buddle, who urged Sir Humphry, to take out a patent for his invention. And we are inclined to agree with Dr. Davy, that, had such a patent been the means of insuring an uniform and correct construction of the lamp, it would have been a most benevolent monopoly. The accidents that have occurred have principally resulted from the parsimonious temerity of coal-owners, employing improper instruments made by ill-informed men. But the refusal of Sir Humphry to take out a patent was a noble one.

"I felt," says Mr. Buddle, "that he did not contemplate any pecuniary reward; and in a private conversation, I remonstrated with him on the subject. I said, 'You might as well have secured this invention by a patent, and received your five or ten thousand a-year from it.' The reply of this great and noble-minded man was—'No! my good friend, I never thought of such a thing: my sole object was to serve the cause of humanity; and if I have succeeded, I am amply rewarded in the gratifying reflection of having done so.' I expostulated (Mr. Buddle continues), saying his idea was much too philosophic and refined for the occasion. He replied, 'I have enough for all my views and purposes: more wealth might be troublesome, and distract my attention from those pursuits in which I delight. More wealth,' he added, 'could not increase either my fame or my happiness. It might undoubtedly enable me to put four horses to my carriage; but what would it avail me to have it said that Sir Humphry drives his carriage and four?" 211.

On the 26th of May, 1818, he quitted England on a second continental journey, undertaken chiefly on account of two objects—the extending the use of the safety-lamp, and the hope of benefiting literature by attempting by chemical means the unrollment of the Herculaneum MSS. In the latter he met with but indifferent success, and he occupied himself, *en passant,* with a theory of volcanoes and some researches into their products. On his return next Spring, to London, he was caught by learning that Œrsted had ascertained that the voltaic pile is a powerful magnet. On this hint, he spoke and worked, and his acuteness and vivid apprehension seized almost instantaneously on many of the laws of electro-magnetism.

Another epoch in his life was at hand. On the 19th of June, 1820, Sir Joseph Banks, who had been for 42 years President of the Royal Society, died. Davy immediately came forward as a candidate for the vacant office. Setting aside the excusable, nay laudable motive of ambition to occupy the place which had been filled by Newton, he conceived that his powers of usefulness would be increased, that he should be able to give an impulse to science, and forward its advancement by example and exhortation; and he flattered himself that he might be able to prevail with the members of his Majesty's government to afford to science some substantial support worthy of the cause and worthy of the country, which to the resources of science had hitherto owed so much and contributed so little. Opposition, though attempted, was fruitless; on the 30th of November, Davy was almost unanimously elected; and, for seven years afterwards, he was annually re-elected.

"I was with him the whole of the day of his first election, and can record with pleasure how tranquilly he passed it: in the morning he had no apprehension of failing of success, and in the afternoon he showed no undue exultation in having obtained it. Before going to the public dinner of the society, held on the anniversary of the election of its officers, he prepared an address, and the speeches it would be required of him to make, as was always his custom when he had to speak in public; for he held that preparation was necessary to speak well. The dinner was very fully attended; and the manner in which his speeches were received, for so grave a body, was quite enthusiastic." 265.

Much scandal has been current with reference to Sir Humphry Davy's bearing in the chair of the Royal Society, or rather as its President out of the chair. It has been said, perhaps by his enemies (and who has not *them?*) that he played the man of fashion, and attuned his tongue to courtly airs, rather than to the sober and democratic accents of science. The following account of his soirées does not seem to give countenance to the injurious imputation.

"The meetings of the Society this year were unusually well attended. Some of the Fellows who had withdrawn during the time of Sir Joseph Banks, owing to the angry contentions which took place in the early period of his presiding, now resumed their attendance; and general harmony and apparent satisfaction prevailed amongst a body of men, so numerous, and of such different tastes and pursuits in literature and science, that it is too much to expect they will consider their interests the same, or even the interests of science, and be for any length of time contented with any President. As it had been the custom of former Presidents to observe a certain state in all that related to their office, conceiving, no doubt, that it helped to maintain its dignity and respectability, my brother did not depart from their example, and he continued to take the chair in a full court dress; to have the splendid mace of office placed on the table before him,* and to sit covered.

His predecessor had for many years an evening party at his own house, for the purpose of assembling men of science, and affording them an opportunity of meeting each other at a fixed time and place. My brother continued this practice, changing only the evening of the meeting from Sunday to Saturday; the former appearing objectionable to some individuals, and he preferring one to which no objection could be made, especially of the serious kind, of interfering with a day which should be set aside for devotional purposes. These evening parties were very similar to those of Sir Joseph Banks; and as long as I was in England, they were numerously attended, and were very agreeable, amusing, and useful. They brought together, not merely men of science, but also literary men, poets, artists, country gentlemen, and they were very attractive to foreigners. The subjects of interest of the day were there discussed, and curious information obtained from the best sources, and knowledge exchanged between individuals, as in a great mart of traffic, each giving and receiving according to his acquirements and wants. There the physiologist and naturalist might collect curious particulars from an African traveller, or Arctic navigator, respecting many objects of his particular inquiries, and give hints for further investigation, or solve questions which might have perplexed the original observers. An evening seldom occurred without some novelty in art, science, or nature, being brought forward—as the bones from the Kirkdale Cave, or a new chemical compound, or a magnetical experiment, or a recently discovered mineral, or some new instrument or apparatus; and a great zest was given by the presence, as was generally

* "It belonged to the Republican House of Commons,—'the bauble' removed by order of Cromwell."

the case, of the inventor or discoverer, who was always willing to offer explanation, and give detailed information to those who were desirous of receiving it. And, moreover, a stimulus was thus imparted—a fresh excitement to the mind to continue and perfect useful investigations; and aids were often given which greatly contributed to the successful termination of scientific labours. In these parties, the distinctions of society seemed very much to be lost in the distinctions which science and merit confer. Men of the highest rank in the country mingled with men without any claim to notice, excepting that high one of superior knowledge; and it was a noble thing to see how much more attractive it was, and more honoured, than the highest nobility destitute of this qualification. I remember one evening, when the company was reduced to a small number by the lateness of the hour, and those who remained had collected round the fire, one of the party, I believe it was Dr. Young, observed in playful remark, 'Ah! I perceive all here are doctors:' and so it proved; there being two or three doctors of physic—one, I believe, of divinity, and three of civil law; and of these last two were baronets, and one was an earl, who, though distinguished for his high bearing on ordinary occasions, on this occasion seemed pleased to be considered of the same grade as the rest." 269.

At this time, Sir Humphry resided in Lower Grosvenor Street, No 28; and as long as he remained in that house he continued to give these weekly evening parties during the session of the Society. Afterwards, when he removed to 26, Park Street, Grosvenor Square, in 1825, they were discontinued; and, as a substitute, the library of the Royal Society, in its apartments, at Somerset House, was opened on Thursday evenings, after the regular meeting was concluded, where the fellows and visitors could converse familiarly on matters of science. Dr. Davy is not precisely acquainted with his brother's reasons for this step. He remarks that when Sir Humphry first became President, he attached some importance to these social meetings, and it was his wish to have made them as agreeable as possible, and as attractive; and to have opened the drawing-room to them, so that ladies might not be excluded; but this he could not effect; and perhaps it might have failed had it been attempted. The parties would, probably, have gained less in gracefulness, ease, and vivacity, than they would have lost in usefulness, zeal, and interest in matters of science. It is to be feared that they would have become fashionable assemblies, rather than scientific meetings. So long as his health permitted, he continued to give the dinners which were expected from him as President, to which were invited, principally, the working Fellows of the Society. The plate which was used on these occasions was very appropriate, consisting chiefly of honorary plate, and principally of the handsome service which was presented to him in 1817, by the great proprietors of collieries in the North of England.

Sir Humphry commenced his presidential duties with high hopes and his wonted enthusiasm. Whether he was, himself, in any degree answerable for his disappointments, we shall not pretend to guess. There is always a sufficiently strong under-current of jealous falsehood and malignity, to baulk the *happiness* of any public man, and perhaps he escapes with least annoyance and least hurt, who possesses most phlegmatic coldness, and aims rather at correctness than at excellence. Enthusiastic temperaments, if qualified for eminence, pay the heaviest tax upon it, and the very ardour that carries them past common men in the right direction, is apt to lead them too far in the wrong. Davy was evidently no man of the world, and possibly his consciousness of his great abilities and his remarkable good fortune, may have led him to expect more from society than it will give. The man who had met with no considerable disappointments, was probably ruffled by slight ones, and at last he took refuge from a world which ill-contented him in one of imagination and of dreams. This conviction cannot fail to press itself on the reader of "The Last Days of a Philosopher." Dr. Davy admits, that his brother did not find the Presidency what he had anticipated, and accounts for it in a not unsatisfactory way :—

"Whilst he was in office, the reputation of the Society was certainly not diminished, but was rather exalted ; the desire to belong to it was increased ; its Transactions were scarcely at any former time more original or interesting ; and at no former period was there more harmony in the general body of the Fellows. And yet, I believe, my brother's expectations were not answered, and he effected very much less than he wished. Government was lukewarm or indifferent in matters of science, and gave him no effectual support; when requiring the aids of science, and of the fellows of the Royal Society, applying to him without hesitation, and, when their objects were attained, forgetting the services. It was his wish to have seen the Royal Society an efficient establishment for all the great practical purposes of science, similar to the college contemplated by Lord Bacon, and sketched in his New Atlantis : having subordinate to it the Royal Observatory at Greenwich, for astronomy ; the British Museum, for natural history, in its most extensive acceptation ; and a laboratory founded for chemical investigation, amply provided with all means requisite for original inquiry, and extending the boundaries and the resources of this most important national science. I remember well his speaking to me more than once on the subject. He had even the idea of raising the funds necessary for forming a laboratory by subscription amongst the Fellows themselves, without the aid of government; and he probably would have attempted this and some other plans for the advancement of science, had his health remained firm.

As regarded satisfaction and pleasure to himself in his official situation, I fear he was much disappointed, and particularly latterly, when he was least able to bear annoyances. He had no idea of manœuvering or managing, and never shrank from responsibility. On him fell the odium of all measures which hurt the feelings of individuals, whether in consequence of the rejection of a paper which the author supposed was worthy of a place in the Philosophical Transactions, or the black-balling of a candidate, ambitious of becoming a Fellow, and, of course, considering himself deserving of that distinction. As no wound, perhaps, rankles more, and is more vexatious than that of personal vanity, so no class of people are more harassing and annoying than those thus offended ; and it is from these that a President of the Royal Society is most exposed to attacks,—persons commonly without any dignity of character, and generally without real ability, feeble, and consequently irritable. The man of real ability, or of true dignity, is above the Royal Society, and need not condescend to resent any act of injustice towards him, supposing the decision of the President and Council to be unjust. He has the world for his tribunal ; and it is only necessary for him to publish the results of his inquiries, and he is sure to have justice done to him. Another source of annoyance, belonging to the office of President is that of the perpetual interruption of his leisure from applications by letter and personally, without end, respecting trifling inventions, supposed by their authors to be important discoveries, respecting patents and certificates for patents, and about imaginary discoveries and schemes worthy of Bedlam, and generally proposed by men of unsound, and often insane mind. To be thus deprived of time, and to have attention and patience wearied, must have been disagreeable to any man, excepting of a trifling character, and to my brother it was particularly very wearying, and it even interfered with his own pursuits, and deprived him very much of the leisure which he might have devoted to original research. As an honorary situation, without profit or emolument of any kind, but occasioning considerable expense to the individual, a stranger to the nature of its duties would suppose the office of President of the Royal Society, for a man of science not only the most elevated, but the most agreeable possible. It undoubtedly should be so; but it never can be so, as long as pretension to knowledge, vanity, and presumption, are more common (and they will always be more intrusive) than real knowledge, modesty, and diffidence. The pleasures of office, and especially of honorary office, are generally in anticipation and imaginary—the trials and troubles, real and incessant." 273.

Nothing is more true than this. But one man's experience is seldom of use to another, not very often, indeed, to himself. Declamations against ambition, and moralisings on the vanity of human wishes, have probably never prevented one ambitious struggle, nor checked one wish.

But although his experiments did not prove successful in reference to the point to which they were ostensibly directed, the establishment of the principle of metallic protection was calculated to prove of extreme utility. Davy himself remarked with perfect truth, in his last Bakerian lecture, *that*, whenever a principle or discovery involves or unfolds a law of nature, its applications are almost inexhaustible; and, however abstracted it may appear, it is sooner or later employed for the common purposes of the arts and the common uses of life.

In the Summer of 1824, he made a voyage to the North Sea, and we have an interesting diary of his excursion. He was not much of a sailor. He makes the following memorandum of Berzelius.

"*Berzelius* was the worthy countryman of Scheele, and certainly one of the great ornaments of the age. Indefatigable in labour, accurate in manipulation, no one has worked with more profit. His manner was not distinguished, his appearance rather coarse, and his conversation was limited much to his own subjects." 319.

But a cloud, the heaviest and blackest that had yet impended over Davy, was now lowering on him, and the brilliancy of his career was to be for ever dimmed. In the Autumn of 1825, not only did he experience some bodily indisposition, but his spirits flagged and their elasticity, for the first time, was seen to give way. He passed an ailing Winter, only to enter on a more ailing Spring. His mother died in September, 1826, and, soon after this, the deterioration in his own health was marked. The following account, furnished by his brother, will be read with interest.

"He experienced more frequently troublesome symptoms, such as uneasy feeling and slight numbness of the right hand, and sometimes pain of the fore-arm, shooting up to the chest; with occasional inordinate action of the heart, and occasional pain and weakness of the right leg. By one of his medical advisers, the pain and numbness of the hand and arm, which on the whole were the predominant ailments, were attributed to an old sprain of the wrist; by another his indisposition was referred to increased flow of blood to the head; and by a friend, a physiologist, to weakness of the heart. He was rather disposed to follow the advice which was most agreeable to the convivial epicurean habits of London Society, and adopt a strengthening diet, as it is called, of animal food, than an abstemious regimen. For some time he ate meat three or four times a day; but he did not improve. When he delivered that discourse which was his last to the Royal Society, at the anniversary meeting on St. Andrew's day, 1826, it was done with such effort that drops of sweat flowed down his countenance; and those who were near him were apprehensive of his having an apoplectic seizure; and he was afterwards so much indisposed as to be unable to attend the dinner of the Society. That day he had the honour of being elected President the seventh time.

When I returned to England in December of the same year, I went to see him at the house of his friend, Mr. Watt Russel, in Northamptonshire, where he was on a visit. He looked well, but stouter than when I left him on my going abroad four years before. He complained of his hand and foot, and of general indisposition, yet he took exercise, and went out with his gun; and he was still on a diet chiefly of animal food, and on a large allowance, his appetite not being bad. We travelled together to London, and in a few days parted to go into different parts of the country.

About a fortnight after, when I was at Hilstone House in Monmouthshire, I had a note from him, dated London, begging me to come to him as soon as I conveniently could, for he was ill. In less than two days I was with him, and I found him much worse than I expected, and labouring under a paralytic attack, affecting the right side. It had come on suddenly while shooting at Lord Gage's. On his arrival in town, to which he hastened, he had put himself under the care of his old and kind friend Dr. Babington,* and of Dr. Holland. The medical treatment employed appeared to have had little or no beneficial effect. As he gained strength, however, the symptoms gradually diminished, and we were very sanguine that he would recover completely." 326.

We observed that the account would be read with interest. We feel for Davy, and we also feel for the mistake that was so obvious, and, perhaps, was so disastrous. Had another view, more correct, and we might add, more rational, been taken, Davy might have been still spared to us, and reserved for fresh discoveries. But, unfortunately, the same blunder that was made in his case, is daily made in others, and while organic lesions are stealing on the brain, the phantom of debility is still invoked, and the willing patient is coaxed and crammed with what he most likes and what is most pernicious.

Davy, though halt in body was active in mind, and pursued some light literary labours. But a journey to the Continent, undertaken in January, 1827, was more likely to conduce to his recovery. He went to Ravenna where he remained for the Spring. His time there, was chiefly spent in taking exercise, in reading, and conversation. About eleven o'clock he commonly got on horseback, and with his gun and dogs, either wandered through the beautiful and extensive avenues of the Pineta, then exhibiting the first burst of Spring, or followed the embankments of the marshes of La Classe, in quest of his favourite petzardone; or, if disinclined for horse exercise, walked on the ramparts of the city. Reading, conversation, and écarté filled up the evening. The reading he then preferred was Lord Byron's poems, of which we had procured a convenient travelling copy, in

* "Babington, the best and warmest-hearted friend, the kindest husband and father, and perhaps the most disinterested physician of his time; with good talents, and a fine tact, and a benevolence which created sympathy for him wherever he appeared, and I believe often cured his patients."—*MSS. Sketches of Contemporary Characters.*

one volume, at Calais. The place gave additional interest to these poems: he had there met their noble author and the lady of his love, under whose influence the muse of Byron had made some of her best efforts; and at that time this amiable and talented woman was at Ravenna residing with her family, and occasionally honoured the invalid with a visit. In April, he shunned the heat of Italy, and sought his old haunts in the Eastern Alps. "Many parts of his journals," says Dr. Davy, "are to me very affecting, as, when recording his wretched health and often miserable sensations, during the struggle he was unremittingly making by all possible means to get rid of his ailments, 'Valde miserabilis!' is not an unfrequent expression; and commonly accompanied with mention of diminished power of limbs and general feebleness, with pains and numbness of limbs. Sometimes he is in despair of recovery, and resigned to his fate; at other times indulging in hope, thankful for feeling better, and expressing thanks (and he does it very often) by the use of letters, such as G. G. D., O. O. O., or G. O. O. D." It would appear that Sir Humphry was, at this time, alone. Dependent, observes his brother, entirely on his own resources; no friend to converse with; no one with him to rely on for aid, and in a foreign country, without even a medical adviser; destitute of all the amusements of society; without any of the comforts of home—month after month, he kept on his course, wandering from river to river, from one mountain lake and valley to another, in search of favourable climate; amusing himself with his gun and rod, when sufficiently strong to use them, with 'speranza' for his rallying word. Now, considering that he had lately been threatened with apoplexy, and was actually labouring under paralysis, we cannot look on it as a judicious plan to have allowed him to wander by himself among the Alps. The "strength of mind" which accomplished this was not accompanied with prudence. His own letter to Mr. Gilbert, in which he resigned the Presidency of the Royal Society, will be an ample commentary on his state and our remark: "I am sorry to say that the expectations of my physicians of a complete and rapid recovery have not been realized. I have gained strength under the most favourable circumstances, very slowly; and though I have had no new attack, and have regained to a certain extent the use of my limbs, yet the tendency of the system to accumulate blood in the head still continues, and I am obliged to counteract it by a most rigid vegetable diet, and by frequent bleedings with leeches and blisterings, which of course keep me very low. From my youth up to last year, I had suffered more or less from a slight hemorrhoidal affection; and the fulness of the vessels, there only a slight inconvenience, becomes a serious and dangerous evil in the head, to which it seems to have been transferred." How can we wonder at Davy's affection. Constant excitement, for, with him, both work and amusement were excitement, produced determination to the head, the earlier symptoms were disregarded, their successors were mismanaged, and then the evil was irreparable.

Almost the last memorandum made on this occasion, or, rather, the last quoted by his biographer, expresses Davy's sentiments on self-destruction:—

"As I have so often alluded to the possibility of my dying suddenly, I think it right to mention that I am too intense a believer in the Supreme Intelligence, and have too strong a faith in the optimism of the system of the universe, ever to accelerate my dissolution. The laurel water, therefore, which I have carried about with me, and used constantly, and from which I have decidedly derived benefit, is a prescription of Tomasini's; and the laudanum and opium which are in my dressing-case, but which *I have never used*, were recommended to me in small doses to remove irritation, taken with purgatives. I have been, and am, taking a care of my health which I fear it is not worth; but which, hoping it may please Providence to preserve me for wise purposes, I think it my *duty*.—G. O. O. O." 381.

He returned to England in October, only to leave it for the last time, in March. In England, he fished and shot when he could, (when did he not?) and we are tempted to give his brother's portrait of the angler, in his best days. This was his attire.

"It was not unoriginal, and considerably picturesque—a white low-crowned hat with a broad brim; its under surface green, as a protection from the sun, garnished, after a few hours' fishing, with various flies of which trial had been made, as was usually the case; a jacket either grey or green, single-breasted, furnished with numerous large and small pockets for holding his angling gear; high boots, water-proof, for wading, or more commonly laced shoes; and breeches and gaiters, with caps to the knees made of old hat, for the purpose of defence in kneeling by the river side, when he wished to approach near without being seen by the fish: such was his attire, with rod in hand, and pannier on back, if not followed by a servant, as he commonly was, carrying the latter, and a landing net. In fishing, as well as in every thing else which he undertook, he displayed extraordinary zeal and energy. It was not unusual for him to go two or three hundred miles for a day's fishing, and his perseverance was in the same proportion. I remember fishing with him from early dawn to twilight in the river Awe in June, for salmon, with little interruption, without raising a fish." 385.

It seems, at first sight, odd, that a mind so ardent as Davy's should take such intense delight in a sport so passive. But, independently of the exercise of *fly* fishing, there is the excitement, the suspense, the hope in angling, which makes it a sort of game of chance, a kind of *water-gambling*. However, an ardent mind must be dashed with the contemplative to enjoy angling thoroughly, and, ardent as he was, Davy loved the beauties of Nature, and was happier far by the river side or on the heather, than with the president's mace of the Royal Society before him, or the fashionable *Blues* of the Royal Institution around him.

He went once more to the Eastern Alps, accompanied by Mr. now Dr. Tobin, and was "disposed to think, with Dr. Philip and Charles Bell, that the radical evil in my case is diseased sensibility in the nerves of the digestive organs, affecting by sympathy the whole sensorial system, and that the determination of blood to the head is only a secondary symptom." His old taste was come on him, for composition of a visionary cast, and besides writing and re-writing his "Salmonia," and his "Consolations in Travel," he planned his Memoirs, and "the Modern Socrates," the design of which is "against all plans of vague ambition; for the simpler pleasures of our nature; and for utility—against war, states-craft, &c., for angling, shooting, the sciences,—against the glory of lawyers, warriors, &c." How changed from the Humphry Davy who took glory by assault with the galvanic battery, and wedded an earthly goddess. In one of his letters, Davy makes rather a whimsical remark, whimsical, at least, for him, for it is one of every-day occurrence. "Poor Dr. Wollaston," he writes, "has an attack of paralysis, and I am sorry to hear is without hopes. His severe and ascetic life has not preserved him. This complaint is certainly becoming more common in England. I have heard of two or three other friends who have likewise suffered,—spare, abstemious men; James Macdonald is one of them."

When a man has anything the matter with *himself*, he fishes out similar cases, and whether it be a cancer or a corn, he soon finds fellow-sufferers, and hugs himself with the consolation that the disease is grown wonderfully common. The philosopher, it will be seen, was no proof against this species of self-flattery. Davy did not know, for he was little of a practical physician, that paralysis, that is, morbid affections of the cerebral vessels and structure, is the natural breaking up of the machine. The old, whether in years or in constitution, may almost lay their account to undergoing it, and even asceticism cannot ward it off. Those who lead a life of mental excitement, those who fill their vascular system to repletion, and those who impair their powers by debauchery, are more prone to suffer than the moderate. But even the moderate cannot escape, and, when death flings his net, the victims past the prime of life may look for "paralysis" as one of its most inevitable meshes.

Davy's end was nigh. Organic disease of the heart and of the head, for who can doubt that he had one or both, was surely, however slowly, doing its work.

On the 20th of February, writes his biographer, most unexpectedly, for there had been no premonitory symptoms, and quite suddenly, he had that severe attack which ultimately proved fatal. That morning (as he told him afterwards) he felt better than usual,—his pulse about 68°; the tongue clean; the ordinary functions of the body well performed. After breakfast he had sat some time, dictating an addition to the sixth Dialogue; when he had finished it he attempted to rise to go into his bed-room, which was adjoining; but found that he could not stand, and that he had lost all power over his limbs, without pain of head, or vertigo, or loss of power of intellect, accompanied merely by a feeling of sickness of stomach. Medical aid was immediately had; leeches were applied to the temples, as if the brain had been affected; and a lowering (or, as it is called, antiphlogistic) plan of treatment was pursued, but with no good effect. He spent the night very restlessly, and the following morning the right side was quite powerless, and the stomach much deranged. He sent for his brother; who got to Rome on the 16th of March. Davy was bad enough, but he was thinking of science. The subjects which now interested him most were, the peculiar electrical power and anatomical structure of the torpedo—his last "Dialogues;" and the second edition of "Salmonia." On these subjects he spoke fully, especially on the first. So Dr. Davy dissected for him, and read to him. More than once there was that final leave-taking, the mournful etiquette of death, and on awaking after a night of such despair, he actually made experiments to satisfy himself that he was alive. This gave his mind a new turn, hope revived, and he "dreamed of bright days to come." When melancholy he had listened to Moore's "Epicurean." Now he preferred Shakspeare's plays; especially his comedies; and the "Arabian Nights," and "Humphrey Clinker." He delighted in hearing these read; and he was not comfortable unless some one was with him almost constantly during the day reading to him. He rallied so much that he drove out, and his passion for the fresh air was as strong as ever. On the 30th of April he could quit Rome on his way to Geneva, where he arrived on the 28th of May, the last *day*, he was to see. His last moments, for time was now to be measured by moments, are painfully interesting. "On our arrival at the inn he merely reclined on a sofa, and occasionally walked to the window, and looked out upon the lake, and expressed a longing wish to throw a fly, as he had been before in the habit of doing, on its waters and on his favourite Rhone. Here he learnt the death of his old friend, Dr. Thomas Young, as I have elsewhere observed.* I was not present when Lady Davy made the communication to him; but when I returned I saw him affected, and he told me how deeply he had been affected by it, even to profusion of tears, and in a manner that was almost unaccountable. In a short time he recovered his composure, and conversed on indifferent matters.

At five o'clock he dined at table, and made a tolerable dinner. After dinner he was read to, according to his custom. At nine o'clock he prepared to go to bed. In undressing, he struck his elbow against the projecting arm of the sofa on which he sat. The effect was very extraordinary; he was suddenly seized with a universal tremor; he experienced an intense pain in the part struck, and a sensation, he said, as if he were dying. He was got into bed as soon as possible. The painful sensations quickly subsided, and in a few minutes were entirely gone. There was no mark or hurt on the elbow, no pain or remaining tenderness; and the effect of the blow perplexed him no less than it did me. A slight feverish feeling followed, which he thought little of, he took an anodyne draught of the acetate of morphine, and then desired to be read to, that his mind might be composed to sleep by agreeable images.

About half-past nine he wished to be left alone, and I took my leave of him for the night, and for ever on earth. His servant, who always slept in his room, called me about half-past two, saying he was taken very ill. I went to him immediately. He was then in a state of insensibility, his respiration extremely slow and convulsive, and the pulse imperceptible. He was dying; and in a few minutes he expired."

No post-mortem examination of his body was made, at his own request: and for how singular a reason! He had a dread of a *post-mortem* examination, founded on an idea which occurred to his mind, that it was possible for sensation to remain in the animal fibre after the loss of irritability and the power of giving proof to others of its existence.

Before we part from Davy altogether, we must not forget to communicate some of those personal traits, and characteristics of sentiment or feeling,

---

* "My brother, in his sketches of the characters of his distinguished contemporaries, thus notices Dr. Young:—

"I must not pass by Dr. Young, called Phenomenon Young at Cambridge; a man of universal erudition, and almost universal accomplishments. Had he limited himself to any one department of knowledge, he must have been first in that department. But as a mathematician, a scholar, a hieroglyphist, he was eminent; and he knew so much that it is difficult to say what he did not know. He was a most amiable and good-tempered man; too fond, perhaps, of the society of persons of rank for a true philosopher."

which connect the hero with common humanity, and are, we believe, almost equally interesting to the reader of cultivated or uncultivated mind.

"He was of middle stature, about five feet seven inches high; but appeared shorter, perhaps from the just proportions and symmetry of his make. His hands and feet were small, and his bones in general small; but his muscles were comparatively large, especially of the lower extremities, in consequence of which he was well adapted for those exercises and sports of the field and river in which he delighted. He could walk well, and bear fatigue for a long time; his arms and shoulders were, he used to say, less able than his legs; yet their strength was perfectly adequate to the management of the salmon rod, and the laborious amusement of salmon fishing; and there were few anglers who could throw the fly further on the water, or with greater steadiness and delicate precision; and he was quick in the use of his gun, and amongst good shots a very tolerable one, especially in that kind of shooting which requires an active hand and eye, as snipe shooting. His chest was well formed and rather ample, and his breathing perfectly good, and he was a good swimmer; yet in early life, as noticed by himself in his 'Researches,' his respiration was unusually rapid, twenty-six in the minute, which is about six above the average; people in health generally making twenty respirations in the minute. As he grew older this quickness of breathing diminished; and latterly I believe it was rather slower than is usual. His neck was rather long and slender: his head was rather small, its surface smooth and rounded, without any striking protuberances; the occipital part was small, the forehead ample and elevated, and very beautifully rising, wide and gently arched. His face was oval, and rather small; but, owing to the expansion of forehead, not apparently so. His features were not perfectly regular; the nose aqualine, and broad at its base; the mouth rather large, the under lip prominent and full; the teeth not large, but irregular; his eyes were light hazle, and well formed; his hair and eyebrows were also light brown; the latter were scanty, the former abundant, and very fine and glossy, with a tendency to curl. I remember once a gentleman speaking to me about it, and expressing his admiration of its quality, very much in the manner he might use in speaking of a lady's hair. His skin was delicate, and his complexion fair, with a good deal of colour. His countenance was very expressive, and responsive to the feelings of his mind; and when these were agreeable, it was eminently pleasing, I might say beautiful, for his smile was so; and his eyes were wonderfully bright, and seemed almost to emit a soft light when animated. His voice was full-toned and melodious, with something in it which impressed his hearers, and made it remembered; indeed, I have heard a lady, who resided in a distant part of the country, and who never saw him, remark, that she hardly ever remembered his name being mentioned without some notice of his voice being made. It was particularly well adapted to express feeling, that kind which was predominant in him,—the high and poetical,—and equally well adapted to convey tenderness and kindness. Without a musical ear, or a quick perception of the difference of sounds, he had studied its intonation carefully, and had so acquired a manner which a person with a fastidious taste for music might find fault with, and yet was very agreeable to a mixed audience. I recollect at the first anniversary dinner of the Royal Society, at which he appeared in his capacity of President, after the cloth was removed, and he had addressed the company in a speech which was extremely well received, the gentleman who sat next me (and who was not aware that I was his brother), turned to me and said, he was sure the President was not musical; that his voice was very fine, but it was deficient in just musical modulation. The person who made this remark was, I believe, an amateur musician, and a distinguished critic in the science of sounds. His senses generally were acute, and well fitted for active life, and the successful pursuit of physical science, in which they are the messengers of information, and unless quick and accurate, may retard and lead astray even the most correct and penetrating minds." 444.

He had a moderate relish for art, an ardent love of Nature. Of sanguine temperament, difficulties stimulated, rather than daunted him, and lasting depression he struggled against, both constitutionally and on principle. As an instance of this his brother cites his conduct in illness. He always strove and attempted to make head against it, trying various remedies, consulting successively different physicians, using different kinds of diet, shifting his abode from the north to the south, according to the seasons and his sensations. Persons who did not comprehend his mind and temperament might misinterpret many of his actions, and especially while he was in a valetudinary state, and attribute almost to derangement of intellect what was the result of activity of mind and unyielding disposition; as his exercising himself not only in walking, but occasionally in running, when he was struggling with a paralytic affection of his right leg, on the idea that the muscles might probably recover their tone by exercise; and in his continuing to shoot and fish at a time when most men would have been confined to their rooms; and in prosecuting his scientific pursuits and literary labours even during dangerous illness, and when he supposed he had only a few hours to live.

He was warm in his friendships, disinterested in his conduct, liberal without profuseness in his expenditure, and moderate in his opinions. Mr. Warburton, it seems, called him, in the House of Commons "a great Tory." This imputation is repelled by his biographer, who cites his advocacy of Catholic Emancipation, his sentiments on the dependence of governments upon the people, and his belief that England holds her pre-eminence among the nations, mainly because her free institutions promote the liberties of man. He would appear then, not to have been "a great Tory," nor a friend to "radicalism, or low democracy." In fact, his leanings were probably rather towards aristocracy, than mere Toryism, terms which, it is well known are not altogether synonymous, Whigs being often most aristocratic.

He was of a social, it would not be going much too far to style it a convivial, disposition, preferring good society, when he could get it, and, when he could not, taking up with worse. His brother tells us of two young officers who accidentally fell in with him on the river side when angling, and spent the evening with him at an adjoining inn, where they stopped for the night. They found their chance companion so singularly amusing, and such "a very good fellow;" so copious in good stories; so knowing in the art of angling and in all things relating to rivers and fish, and to the gun as well as to the rod,—that their curiosity was excited to inquire of the landlord who he was; and their surprise was great on being informed that he was the President of the Royal Society. That he was ambitious of distinction in conversation is pretty evident, from his ardour when well, his seclusion when ill. We have heard it said, that he was too diffuse and too flowery, both in speaking and in lecturing, and his brother virtually confesses it. The man who was fond of good society would be likely to be fond also of good dinners and good wines; and Davy was so. Yet he was no free liver.

" In dress he was rather careless, especially latterly; consulting more health and comfort than fashion and appearance. Before the present undress of gentlemen came into vogue, after the termination of the war, short breeches, black silk stockings, a blue coat, and a white waistcoat, and white neckcloth,—the common costume of the time,—was his usual attire; but he retained it no longer than it was common. He was fond of broad-brimmed hats, as they afford protection from the sun and rain, and he generally wore one in travelling. I remember his wearing one of very moderate dimensions when he came to Edinburgh in 1811, soon after his marriage, at the time I was studying there; and in walking through Princes-street it attracted the impertinent notice and remark of some young men who were following us. At that time there was so little intercourse with foreigners, and dress was so uniform, that any small innovation was considered a great peculiarity. 459.

From an animated sketch of him by Mr. Poole, we are tempted to extract one anecdote, which exhibits Davy's freedom from sordid national jealousies.

"Men of science, wherever situated, he considered as fellow-subjects of one great republic, spread over the world. I was in London soon after he received the letter from France, announcing that the National Institute had awarded him the prize given by Napoleon to the greatest discovery by the means of galvanism. (These discoveries are detailed in the Transactions of the Royal Society, 1807.) He showed me the letter, and said, 'Some people say I ought not to accept this, and there have been foolish paragraphs in the papers to that effect; but if the two countries or governments are at war, the men of science are not,—that would indeed be a *civil war* of the worst sort.'—'Rather,' he added, 'we should, through the instrumentality of men of science, soften the asperity of national war.'" 466.

Dr. Davy introduces the characters of his brother, drawn by Dr. Henry, and by Professor Silliman. They are highly laudatory, yet, probably, little more laudatory than just. One passage from Silliman is worth quoting:—"To conclude, we look upon Sir Humphry Davy as having afforded a striking example of what the Romans called *a man of good fortune*;—whose success, even in their view, was not however the result of accident, but of ingenuity and wisdom to devise plans, and of skill and industry to bring them to a successful issue. He was fortunate in his theories, fortunate in his discoveries, and fortunate in living in an age sufficiently enlightened to appreciate his merits;—unlike, in this last particular, to Newton, who (says Voltaire), although he lived forty years after the publication of the *Principia*, had not, at the time of his death, twenty readers out of Britain. Some might even entertain the apprehension that so extensive a popularity among his contemporaries, is the presage of a short-lived fame; but his reputation is too intimately associated with the eternal laws of Nature to suffer decay; and the name of Davy, like those of Archimedes, Galileo and Newton, which grow greener by time, will descend to the latest posterity."

Dr. Davy terminates his task by a defence of contemporary biography. Mr. Babbage had observed, in reference to Wollaston and to Davy, "until the warm feelings of surviving kindred and admiring friends shall be cold as the grave, from which remembrance vainly recalls their cherished forms invested with all the life and energy of recent existence, the volumes of their biography must be sealed; their contemporaries can expect only to read their *éloge*." Dr. Davy rather contends for the propriety of this, and holds that the maxim, de mortuis nil nisi bonum, is not only a generous but just one, intended and calculated to protect the dead from slander. "To hold up," he says, "the infirmities of a man of genius to observation is neither necessary nor useful: on the contrary, injurious, as tending to lower him as an example in the minds of posterity, and diminish the influence of his name. Almost from the nature of biography, it appears to me that it *ought* to be laudatory." This is a gallant view of biography, and worthy of the brother of Davy. But we are not sure, that, to sacrifice truth to the ideal, is productive of so much utility as an ardent mind might anticipate. Exaggeration is suspected, the picture is deemed flattering and false, and scepticism or envy subtracts more than truth would have deducted.

But, after all, the life of Humphry Davy should be read by young and old, to stimulate the one, to console the other. It is the life of a man of high aspirings, a generous spirit, a noble enthusiasm, and a fine genius. His fortune was of his own earning, and never was it gained with more praise and less to blame in the winner.

He loved science more for its own sake, than for that of the power or the dignity it brought; and money, the base divinity to which so many bow the knee, was little worshipped by him. These qualities of mind are admirable, and worthy of all imitation, and cannot be held up too earnestly or too long before the eyes of youth. Nor are the last days of the philosopher without their portion of sad instruction. Rich in wealth and honour and in the opinions of men, possessed of what ambition craves and sacrifices all to get, surrounded with what the poor believe and the great would have us think the appurtenance and means of happiness, Davy found that these ministers of pleasure spoke with lying tongues, and fell back on what is purchaseable at no costly price, a love of nature, a desire for philosophic truth, and utility to mankind for an aim and object. His example then encourages in every way—to the ardent it points out the road to fortune—to those on whom fortune *will* not smile, it gently whispers her insignificance. Whatever might have been the faults of the man, they were as nothing compared with his virtues, and his constant aspiration to do good to humanity, the stimulus of his labours, the consolation of his sickness, have woven an undying wreath to his memory.

---

II. PHILIP SYNG PHYSICK was born in Philadelphia, on the 7th of July, 1768. His father, Mr. Edmund Physick, was an Englishman. Previously to the separation of the United States from Great Britain, he held the office of Keeper of the Great Seal of the Colony of Pennsylvania; and subsequently to the Revolution he took charge of the estates belonging to the Penn family, and served as their confidential agent. Physick's school-boy days present nothing remarkable. In June, 1785, one month after he obtained the degree of Bachelor of Arts, he commenced the study of medicine, under the superintendence of the late Doctor Adam Kuhn, well known as the pupil of Linnæus, and a most distinguished and successful practitioner, and then Professor of the Theory and Practice of Medicine in the University of Pennsylvania. He remained at the University for three years, when, to complete his education, he determined to visit Great Britain, and avail himself of the advantages which were afforded by the great schools and hospitals of London and Edinburgh. He arrived in London in January, 1789, and became a private pupil of John Hunter's. Dr. Physick continued to prosecute his studies with the most exemplary perseverance and industry, under the immediate superintendence of Mr. Hunter, throughout the year 1789. On the first of January, 1790, he was appointed House Surgeon to St. George's Hospital for one year, which is the usual period of that service in the institution. This appointment he owed exclusively to the patronage and influence of Mr. Hunter.

Dr. Physick was fortunate in obtaining such an appointment so early; no amount of interest would now, after only a year's attendance, procure it. Dr. Randolph believes, and no doubt with justice, that it was during the period of his remaining in St. George's Hospital, that Dr. Physick acquired a vast deal of that surgical skill and dexterity which laid the foundation of his subsequent greatness.

"An anecdote frequently related to me by Dr. Physick, connected with his early appointment to St. George's Hospital, I trust I may be pardoned for mentioning here, notwithstanding it has already been promulgated from another source. His success in obtaining this situation caused some slight degree of dissatisfaction on the part of some of the disappointed applicants, who conceived that their claims for the situation were stronger than his. In consequence of this Dr. Physick clearly perceived that they evinced an uncommon share of curiosity respecting the manner in which he discharged his duties, and that they were disposed to scrutinise his actions with the greatest strictness. A short period after commencing his services, a patient was admitted into the hospital who had had the misfortune to dislocate his shoulder; the head of the humerus was thrown downward, and lodged in the axilla. Fortunately the accident was quite recent. It so happened that at the time the man was admitted the whole class were in attendance at the house. They, of course, were exceedingly anxious to witness the manner in which the reduction would be effected, and Dr. Physick was perfectly well aware that his method of restoring the bone to its natural situation would be subjected to severe criticism. He directed the patient to be seated upon a high chair; he then proceeded to examine the injured shoulder very particularly, and questioned the man as to the manner in which the accident had occurred. Whilst making these inquiries he placed his left hand in the axilla, and taking hold of the lower end of the humerus with his right hand, he made all the extension in his power; he then suddenly depressed the elbow of the patient to the side of his body, and in so doing, dislodged the head of the bone, which glided instantaneously into the glenoid cavity, very much to his own delight, and doubtless also to the perfect satisfaction of the class." 25.

In January, 1791, after giving up his house-surgeoncy, he received the diploma from the London College of Surgeons, and Mr. Hunter's sense of his merits was evinced in a very decisive manner. He invited him to take up his residence with him, to become an inmate of his house, and to assist him in his professional business; he also held out inducements to him to establish himself permanently in London, and to pursue the practice of his profession in that city. But Physick declined the offer, and merely remained with Hunter till May, when he proceeded to Edinburgh, where, in May, 1792, he obtained the degree of M.D.

He returned to his native country in September, 1792; and commenced the practice of his profession in Philadelphia. His manners and appearance were eminently in his favour. Dr. Randolph tells us that "he was of a medium height; his countenance was noble and expressive; he had a large Roman nose; his mouth was beautifully formed, the lips somewhat thin, and he had a high forehead, and a fine hazel eye, which was keen and penetrating. The expression of his countenance was grave and dignified, yet often inclined to melancholy, more especially when he was engaged in deep thought, or in performing an important and critical operation. Dr. Physick rarely indulged in excessive mirth; he was, however, far from being insensible to playful humour, and on such occasions his countenance would be lighted up by a benign smile, which altered entirely the whole expression of his features. His manners and address were exceedingly dignified, yet polished and affable in the extreme; and when he was engaged in attendance upon a critical case, or in a surgical operation, there was a degree of tenderness, and at the same time a confidence, in his manner, which could not fail to soothe the feelings and allay the fears of the most timid and sensitive.

Dr. Physick, as might be well supposed, was not oppressed with practice for the first year. But, fortunately for him, the yellow fever afflicted Philadelphia in 1793, and Physick was elected Physician to the Yellow Fever Hospital at Bush Hill. The populace, as is usual during pestilences, being disposed to be riotous, Dr. Physick was created an alderman, for the purpose of helping to prevent disturbance. All this brought him forward, and gained him some valuable connexions. He was the first to promulgate the doctrine that yellow fever was not contagious—his dissections went to establish the existence of gastritis as an essential feature of it—and he was a warm advocate of antiphlogistic treatment. He had an attack himself, which shook his constitution. In the year 1794, Dr. Physick was elected, by the managers of the Pennsylvania Hospital, one of the surgeons to that institution. This period may be stated to be the dawn of his great surgical fame and usefulness; indeed the appointment was the stepping-stone to his fortunes. Surgery was then at a low ebb in America, and a disciple of John Hunter was wanted to reform it. The first thing he did was to improve the treatment of ulcers. He soon made the wards clear of them. His method of treatment in cases of inflamed and irritable ulcers was exceedingly simple. He directed the patient to be confined to bed, and to be kept strictly at rest; and in cases where the ulcer was situated upon a lower extremity, he caused the limb to be considerably elevated. He next directed that mild and soothing applications should be made to the ulcer itself; and in conjunction with this he made use of proper constitutional treatment. In cases where the ulcer partook of an indolent nature, he always preferred effecting the necessary stimulation by means of local applications, whilst the patient was confined to bed, to permitting him to walk about, as sometimes recommended. Perhaps, even now, some parts of his method might be insisted on with advantage. In the year 1794, Dr. Physick was elected one of the physicians to the Philadelphia Dispensary. He subsequently was chosen one of the consulting surgeons to this same institution, and retained the situation till the time of his death.

In 1795, there was a considerable increase in his professional engagements, and his prospects of success in practice were flattering. He now commenced a Journal of his more remarkable cases, which he continued until 1810. It would seem that he operated well for cataract, the extraction of which he preferred; and his biographer is of opinion that his operations upon the eye, in conjunction with those for stone in the bladder, did as much in establishing his great surgical character as any others which he ever performed. In his first operation for lithotomy, in 1797, he accidentally divided with his gorget the internal pudic artery. The hemorrhage from the wounded vessel was exceedingly profuse. He immediately compressed the trunk of the artery with the fore-finger of his left hand, and then passed the point of a tenaculum under it; a ligature was then cast round it and firmly tied. This of course arrested the hemorrhage, but the ligature included along with the artery a considerable portion of the adjacent flesh. In order to obviate this inconvenience Dr. Physick subsequently contrived his celebrated forceps and needle for the purpose of carrying a ligature under the pudic artery.

It appears that Physick was dexterous in the treatment of stricture, and a first-rate maker of waxed bougies. In the year 1795, he invented an instrument which is much used (it may be modified) by some at present, for the purpose of cutting through a stricture which had refused to yield to the ordinary methods of treatment. This instrument consists in a lancet concealed in a canula, which is passed down to the stricture, and then the lancet is pushed forward so as to effect its division.

During the years 1797, 1798, and 1799, the yellow fever again ravaged Philadelphia, and Dr. Physick was again found in the foremost rank of those who had to contend against its ravages. Whilst engaged in the performance of his duties in the year 1797, he was attacked himself for the second time, with the fever, and his illness was so severe that for some time but slight hopes were entertained of his living. His recovery from this indisposition was exceedingly slow, and he was left in such an enfeebled state that he was obliged to go into the country to recruit his strength. During the prevalence of the yellow fever in 1798, Dr. Physick was again resident physician at the Bush Hill Hospital; and upon leaving the institution after the subsidence of the epidemic, he was presented in a flattering manner by the board of managers, with some valuable silver plate, as an acknowledgment of their "respectful approbation of his voluntary and inestimable services."

In 1800, he married Miss Eliza Emlen, by whom he had four children, two sons and two daughters, still alive. In the same year, he was requested by a number of students, in the University of Pennsylvania, to deliver lectures on surgery, which he did, and with success; five years afterwards, the Professorship of surgery was created in the University of Pennsylvania, and Dr. Physick was appointed to fill the chair. In 1802, he published a case of hydrophobia, and recommended tracheotomy. In this year, he performed his operation of passing a seton through an ununited fracture of the humerus. Dr. Randolph communicates a rather interesting circumstance in connection with this case.

"It so happened that, in the year 1830, I was requested to visit a patient in Third street, who was lying dangerously ill from an attack of remitting fever of a high grade. A few days after my first visit, in riding past his door in company with Dr. Physick, feeling very uneasy about the condition of my patient, I requested the Doctor to step into the house and see him with me, and give me the benefit of his advice. He complied with my request, and upon entering the sick man's chamber he immediately recognised him as the individual upon whom he had performed the operation which I have just described, twenty-eight years previously. Upon questioning the patient he informed us that the arm which had been broken was quite as strong as the other arm, and that he had never sustained any inconvenience from the operation. Eventually the man died; and having obtained permission to make a post-mortem examination, I procured his humerus, and still have it in my possession, regarding it as one of the most interesting and valuable pathological specimens extant. At the place of fracture, the two ends of the bone are perfectly consolidated by a considerable mass of osseous matter, in the centre of which there is a hole, showing the place through which the seton passed." 60.

Dr. Randolph assures us that Dr. Physick worked excessively hard. He appears to have been, in this, a worthy pupil of John Hunter, who asked, as our readers may remember, a young man to breakfast at six o'clock in the morning. It was Physick's custom, throughout the winter months, to rise at four o'clock in the morning. This hour being too early to disturb a servant, he was obliged to arrange his own fire. He would then sit down to his desk and prepare his lecture for the day; after which he would dress himself, and then take his breakfast, and leave his house between eight and nine o'clock, in order to attend to a most extensive and laborious practice. In addition to all this, he discharged his duties as Surgeon to the Pennsylvania Hospital, and to the Alms House Infirmary. He used often to remark, that in order to obtain entire success as a practitioner of medicine, it was necessary to work hard. Success was not made a present to him.

His manner, as a Lecturer, his biographer assures us, was grave, dignified, and impressive to an extraordinary degree. His style was clear and comprehensive, simple yet chaste. He was uniformly careful never to say too much. His choice of language was remarkably good, and he possessed the happy faculty of communicating knowledge agreeably and well, in great perfection. Perhaps one great reason for this was, that he never undertook to instruct others upon subjects which he did not clearly comprehend himself. He attempted no display of oratory; neither did he permit his reason and imagination to run wild in the regions of theory and fancy. His lectures were all carefully prepared and written out. He did not at all approve of extemporaneous lecturing; as he thought that in lecturing upon scientific subjects, and more especially such as involved the lives and happiness of our fellow-beings, no man had a right to place so much confidence in the strength of his memory as is implied in that practice. Possibly, Physick may be deemed over-scrupulous in this instance. Lecturers and lectures have so multiplied now-a-days that pupils have become particular, and will not listen to every-body. Written lectures read from books are not much relished, and a little precision must be sacrificed to popularity. Nor need memory alone be trusted to. Notes may be used of sufficient fulness to prevent either omissions or errors of moment, and the little that is lost in minute detail, is more than compensated by what is gained in freedom and effect. Physick was a practical lecturer, *the* great thing.

As a letter-writer he was punctual and peculiar. His letters in general were remarkably brief and pithy. Having said all that he considered necessary for the elucidation of his subject, he invariably stopped. He would reply to a letter of three or four pages closely written, in about as many lines. He was excessively annoyed at receiving, and being obliged to read letters of an unmeaning and unnecessary length. The same thing took place with respect to books. A complaint not confined to Physick.

In the year 1809, Dr. Physick performed successfully that operation for artificial anus, which anticipated the procedure of Dupuytren. Previous to his death, Dupuytren was fully satisfied that Physick had preceded him in the invention.

In the Winter of 1813-14, he suffered from a severe attack of typhus fever, which again shook him severely. From this time, indeed, he never enjoyed good health. He grew subject to dyspepsia, and to frequent attacks of catarrh, and his susceptibility to this condition increased to such an extent that he was obliged to observe the most rigid precautions in order to guard against it. His method of treatment when labouring under a severe cold, required confinement to a warm room; and in fact he accustomed himself to a degree of heat in his apartments which to many others was almost insupportable. In addition to this he always employed the strictest antiphlogistic treatment, as regarded his diet and his remedial agents; treatment, in Dr. Randolph's opinion, far too debilitating, and the cause of his subsequent prostration.

About this period he began to experience certain unpleasant symptoms, indicative of a diseased condition of the heart, and which eventually terminated in organic affection of that organ. And he suffered too from calculous concretions in the kidneys. "It is impossible," says his biographer, "for language to describe the pain and agony which he frequently endured from the passing of the small calculi through the ureters into his bladder. Upon one occasion, about ten years previous to his death, I knew him to be for near two hours without any pulse perceptible at the wrist, in consequence of intense suffering, caused by the lodgment of a small calculus in the ureter. It remained fixed in this situation for some days, and grew to the size of a small pea; it finally passed into the bladder, and was discharged a few minutes subsequently through the urethra." Dr. Physick was an advocate for animal ligatures, which he always employed. He adopted an ingenious contrivance for facilitating the discharge of ligatures which remained fixed in the cavity of wounds, either in consequence of being penetrated by new granulations, or from other causes. In such cases he twisted the ligature very firmly, and then secured it to the adjacent skin, by means of a small strip of adhesive plaster. The effect of this twisting is to tighten the noose at the extremity of the ligature, so as to compress completely the parts contained within it; and in addition to this, the natural tendency of the ligature to untwist itself keeps up a constant action and pressure upon the parts, and thereby causes ulceration.

Physick was at the height of his practice, when infirmities came fast upon him. He resigned his situations in the Philadelphia Dispensary, and in the Alms House Infirmary; and, in 1816, he resigned his situation as surgeon to the Pennsylvania Hospital. In 1819, he resigned the chair of Surgery in the University of Pennsylvania, and was transferred to that of Anatomy; which had become vacant the preceding session by the death of his nephew, Dr. John Syng Dorsey, an event which afflicted him sorely. In 1831, this last situation was too much for him, and he resigned it also. The Institution conferred on him the highest honour that it could, by electing him unanimously "Emeritus Professor of Surgery and Anatomy."

One of Physick's latest contributions to surgery, was an account of preternatural pouches, or sacs, situated at the lower extremity of the rectum, just above the verge of the anus. This form of disease, which is one of not unfrequent occurrence, is in many instances productive of the most severe and distressing symptoms. In 1835, Dr. Physick used an ingenious apparatus in the case of a gentleman aged 70, who had long suffered from an enlargement of the third lobe of the prostate gland. The following is Dr. Dorsey's account of it:—

"The end of a small flexible catheter was introduced nearly to the bottom of a very thin sac or pouch, three inches long, and an inch and a half in diameter at the mouth. The edges of the sac, which was prepared from the intestine of a sheep, were secured to the catheter by a fine silk thread, wrapped around it with great care; and the material being as fine as the thinnest blotting-paper, adapted itself, when oiled, so closely to the instrument, that the bulk of the whole was less than that of a large sized bougie.

After its introduction into the bladder, the membrane was injected with tepid water, and the mouth of the catheter being stopped with a peg, it was gently, but with some firmness, retracted. The consequent pressure at the seat of disease, gentle and uniform, and from the nature of the material used, as little irritating as possible, had the happiest effect in repressing the enlarged lobe of the gland; and afforded, for many months, great relief by facilitating the discharge of the urine. Although the patient took a severe cold immediately after the operation, he did not suffer more than he had previously; and on recovering from its temporary influence, he experienced a relief long unknown. The introduction of the instrument was again practised after an interval of some months, with great advantage.

Much nicety is requisite in securing the edges around the catheter, so that there may be no roughness to cause irritation during its retraction. It was also deemed proper to wind the end of the thread loosely round the catheter and secure it to the stopper. The material employed was prepared and may be procured in France." 105.

Dr. Physick relieved another case by the same means; we confess that we should not expect much from it. The last operation he ever performed was the extraction of a cataract. It was on the 13th of August, 1837. From this time, his complaint went on increasing in intensity and violence. The symptoms of hydrothorax became developed to a most painful extent, and he suffered extreme agony from oppression at his chest and difficulty of breathing; so much so, that sometimes he became unable to lie down in his bed for whole nights together, but was obliged to stand upon the floor, supported by assistants. Some time previously to his death, anasarca took place; and in consequence of his remaining so much in the erect position, his lower extremities became enormously swollen and distended with serum. The integuments at length gave way, openings were formed, and these finally ulcerated and became gangrenous.

The Father of American Surgery expired without a struggle, on the morning of the 15th of December, 1837, at twenty minutes past eight o'clock.

Dr. Physick was, in private life, exemplary—a good man and a good citizen.

"It must be admitted," says Dr. Randolph, "that by the community at large, Dr. Physick's private character was but imperfectly understood. This was owing to the habits of perfect seclusion which he contracted, and to the slight intercourse, other than professional, which he permitted himself to enjoy with his fellow-citizens. It must not be supposed, however, that this isolation arose from moroseness of character or want of inclination to mingle with society. A satisfactory explanation may be afforded by the entire self-abandonment with which he devoted himself to his professional engagements. In my opinion this formed one of the most striking and remarkable points in Dr. Physick's character. I doubt very much whether history could show an example of a more pure and absolute devotion to professional pursuits than he exhibited.

In consequence of the reasons just mentioned, he was supposed by some to be stern and unfeeling, and wanting in the kinder sympathies of our nature. There could not be a greater misapprehension. His feelings were tender and susceptible in the extreme; and could those persons who entertained an opposite opinion have been admitted behind the scenes, and to closer and more intimate relations with him, they would have acknowledged the great injustice they had done him in such a surmise. Many instances might be cited, were it expedient to occupy the necessary time, in order to illustrate Dr. Physick's extreme tenderness of feeling. At an early stage of his professional career, he performed a few experiments upon living animals, with the view of determining some physiological points. This formed a lasting subject of regret to him as long as he lived; and he could not divest his mind of the idea that he had been guilty of a useless as well as a wicked act of cruelty.

Previously to his performing important surgical operations, his feelings were so harrowed up, and he experienced so much anxiety, that it was the custom of his family to endeavour to prevail upon him to execute such operations as speedily as possible, in order to relieve his mind." 111.

Thus we have another example of the indisposition to perform great and bloody operations, experienced by a really eminent surgeon. A noble trait in such minds as those of Cheselden and Physick. We complete the picture of an estimable, as well as a distinguished medical man, when we add, that, in his intercourse with his professional brethren Dr. Physick's conduct was regulated by the strictest principles of honour and integrity. Whenever he was called in consultation with other physicians, without enquiring how exalted or humble their positions might be, he was scrupulously careful to avoid saying or doing anything which could wound their feelings, or prejudice them in the least in the estimation of their patients. He invariably stated his own opinions in a frank and manly manner, and was ever willing to pay due deference to the opinions of others. Upon all occasions he was happy and ready to confer upon his fellow-practitioners the benefit of his advice and experience, whether the information desired had special relation to themselves, or to those under their charge. He was far removed above the meanness of interfering with the patients of others; and whenever he had it in his power to render a service to a younger member of the profession, by a word of encouragement or commendation, it was cheerfully bestowed.

Physick is justly regarded in America, as the father of her surgery. His native land is proud of her son, and the "Old Country" may lay claim to a share in forming his professional character. Possessed of great application, a mind of healthful energy and purpose, and a love for the profession he had chosen, Physick had the qualities of head and heart, which are calculated for success in either hemisphere. The lessons of John Hunter had sunk deep into his soul, and the pupil was animated with a generous ambition, to prove himself worthy of the master. The integrity of the man supported and dignified the ability of the surgeon, and Dr. Physick may properly and usefully be cited, as one of the many models of professional life, that our annals are rich in. It gives us a mournful pleasure to pay this tribute to his memory, and to paint the virtues of this good, and, in his own science, this great man, for the contemplation and the benefit of the youth of America and Britain.

This article has spread to too great a length to permit us to touch upon Mr. Pettigrew's excellent work. In our next number we shall return to it, and as Mr. Pettigrew has brought it to a conclusion, we shall give an account of those lives contained in it which we have not hitherto noticed.

